# The coverage and challenges of increasing uptake of non-National Immunization Program vaccines in China: a scoping review

**DOI:** 10.1186/s40249-023-01150-8

**Published:** 2023-12-08

**Authors:** Mingzhu Jiang, Shu Chen, Xuanxuan Yan, Xiaohua Ying, Shenglan Tang

**Affiliations:** 1 School of Public Health, Fudan University, 130 Dong’an Road, Xuhui District, Shanghai, 200032 China; 2 Australian Research Council Centre of Excellence in Population Ageing Research (CEPAR), University of New South Wales, Sydney, Australia; 3 School of Risk and Actuarial Studies, University of New South Wales, Sydney, Australia; 4 Global Health Research Center, Duke Kunshan University, Kunshan, Jiangsu China; 5 SingHealth Duke-NUS Global Health Institute, Duke-NUS, Singapore, Singapore; 6 Duke Global Health Institute, Duke University, Durham, USA

**Keywords:** Non-National Immunization Program vaccines, HPV, Hib, PCV, Rotavirus, Coverage, Uptake, Barriers, Challenges

## Abstract

**Background:**

Non-National Immunization Program (NIP) vaccines have played an important role in controlling vaccine-preventable diseases (VPDs) in China. However, these vaccines are paid out of pocket and there is room to increase their coverage. We focused on four selected non-NIP vaccines in this study, namely *Haemophilus influenzae* type b (Hib) vaccine, human papillomavirus (HPV) vaccine, pneumococcal conjugate vaccine (PCV), and rotavirus vaccine. We aimed to conduct a scoping review of their vaccination rates and the major barriers faced by health systems, providers, and caregivers to increase coverage.

**Methods:**

We followed the Preferred Reporting Items for Systematic Reviews and Meta-Analyses Extension for Scoping Reviews (PRISMA-ScR). We searched five English databases (PubMed, Web of Science, EMBASE, Scopus, and WHO IRIS) and four Chinese databases using the search strategy developed by the study team. Two independent reviewers screened, selected studies, and examined their quality. We summarized the non-NIP vaccine coverage data by vaccine and applied the 5A framework (Access, Affordability, Acceptance, Awareness, Activation) to chart and analyze barriers to increasing coverage.

**Results:**

A total of 28 articles were included in the analysis (nine pertaining to vaccine coverage, and another 19 reporting challenges of increasing uptake). Among the four selected vaccines, coverage for the Hib vaccine was the highest (54.9–55.9% for 1 dose or more from two meta-analyses) in 2016, while the coverage of the other three vaccines was lower than 30%. Eight of the nine included articles mentioned the regional disparity of coverage, which was lower in under-developing regions. For example, the three-dose Hib vaccination rate in eastern provinces was 38.1%, whereas the rate in central and western provinces was 34.3% and 26.2%, respectively in 2017. Within the 5A framework, acceptance, awareness, and affordability stood out as the most prominent themes. Among the 12 identified sub-themes, high prices, low vaccine awareness, concerns about vaccine safety and efficacy were the most cited barriers to increasing the uptake.

**Conclusions:**

There is an urgent need to increase coverage of non-NIP vaccines and reduce disparities in access to these vaccines across regions. Concerted efforts from the government, the public, and society are required to tackle the barriers and challenges identified in this study, both on the demand and supply side, to ensure everybody has equal access to life-saving vaccines in China. Particularly, the government should take a prudent approach to gradually incorporate non-NIP vaccines into the NIP step by step, and make a prioritizing strategy based on key factors such as disease burden, financial resources, and market readiness, with special attention to high-risk populations and underdeveloped regions.

**Graphical Abstract:**

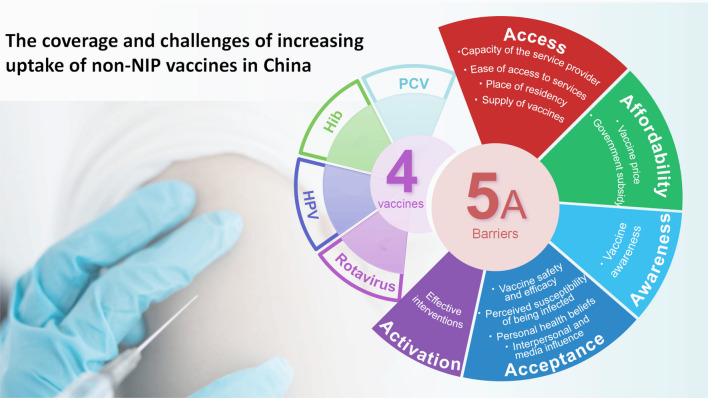

**Supplementary Information:**

The online version contains supplementary material available at 10.1186/s40249-023-01150-8.

## Background

Vaccines are one of the most cost-effective interventions to save lives and improve health and well-being [1]. In China, National Immunization Program (NIP) vaccines are freely administered to eligible children whereas non-NIP vaccines are voluntarily administered and self-funded. Currently, there are more than 30 types of non-NIP vaccines in China, which can be broadly categorized into two main types based on their intended use [2]. The first is alternative non-NIP vaccines, which are designed for diseases already covered by the NIP but differ in characteristics or vaccination procedures. One example is the pentavalent vaccine (DTaP-IPV/Hib), which combines vaccines for diphtheria, pertussis, tetanus, polio, and Hib that are already included in the NIP. The other is supplementary non-NIP vaccines, which prevent diseases not yet included in the NIP, such as the Hib, HPV, PCV and rotavirus vaccine.

Our study focuses on four non-NIP vaccines that are recommended by the World Health Organization (WHO) for inclusion into the NIP of all Member States: Hib, HPV, PCV and rotavirus vaccines [3]. These four vaccines have demonstrated high efficacy, safety, and effectiveness in preventing targeted diseases including invasive bacterial disease (meningitis, pneumonia, septicemia), cervical cancer and childhood diarrhea, and have achieved high coverage globally [4]. A study based on estimates from the Global Burden of Disease (GBD) Study 2017 showed that increased Hib vaccine and PCV vaccine coverage were the largest contributors to decreases in lower respiratory infection mortality among children younger than 5 years between 1990 and 2017, globally [5], and that full vaccination against rotavirus could have averted an estimated 22.0% of deaths caused by diarrhea during the same timespan [6]. Furthermore, after 5–8 years of being vaccinated, the prevalence of HPV 16 and 18 infections decreased significantly by 83% among girls aged 13–19 years, and by 66% among women aged 20–24 years [7]. Among the 194 WHO Member States, 99.0%, 66.5%, 82.5% and 59.8% of them have included Hib, HPV, PCV, and rotavirus vaccines in their NIPs, respectively [8].

Despite the crucial role of the four non-NIP vaccines in disease prevention, their uptake in China remains relatively low in comparison to NIP vaccines. The coverage of all NIP vaccines has remained above 95% since 2012 [9, 10]. Unfortunately, there are no official statistics publicly available on non-NIP vaccine coverage, only fragmented evidence from various studies. For example, one study showed that in some areas with high levels of economic development, the three-dose HPV vaccination rate in Shanghai was estimated to be 2.8% in 2017–2019 [11], and full vaccination of PCV13 among children aged 0–15 months in Jiangsu province was 6.2% in 2019 [12]. The National Immunization Advisory Committee (NIAC) has been working on facilitating the expansion of the NIP to increase the uptake of key non-NIP vaccines [13]. However, despite a limited number of local pilot programs that have included HPV [14] and PCV vaccines [15] in the local immunization programs, these vaccines are still paid out of pocket at a high price in a vast majority of China’s regions.

In the absence of sufficient and systematic empirical data to explain the factors affecting non-NIP vaccine uptake, this study seeks to explore the barriers to vaccine uptake in China through the 5As taxonomy. The 5As taxonomy was developed in 2016 through an extensive review and integration of insights from other models, aiming to comprehensively delineate and categorize vaccine uptake across all non-socio-demographic aspects. The 5As taxonomy tests that individuals could have access to vaccines (Access), be financially capable of affording them (Affordability), be adequately informed about their safety and efficacy (Awareness), willingly accept vaccination (Acceptance), and diligently adhere to the vaccination schedule with appropriate reminders (Activation). Previous research has identified a range of factors contributing to low uptake of non-NIP vaccines in China [16–18], including health beliefs [19], financial constraints [20], and concerns about safety and acceptance [21]. However, the information remains fragmented and lacks a cohesive framework to integrate, identify, and address this complex issue. Given the different socio-cultural contexts and intricate status of vaccination practices in China, adopting the 5As taxonomy can provide a comprehensive and coherent approach to understanding and tackling the challenges associated with non-NIP vaccine uptake.

Advancing and sustaining high and equitable immunization coverage is a global and national priority in achieving the health-related sustainable development goals (SDGs) by 2030 which, among other goals, includes ending preventable deaths of newborns and children under 5 years of age [22]. Both the Immunization Agenda 2030 (IA2030) [23] and Healthy China 2030 goals [24] emphasize extending immunization services to under-immunized children and communities. We conducted a scoping review to synthesize evidence available on the coverage of the four non-NIP vaccines in China, and the major barriers that impede uptake of these vaccines. This review aims to generate robust and synthetic evidence for developing effective strategies to increase the coverage of these selected vaccines in China.

## Methods

### Overview

This study adhered to the Joanna Briggs Institute methodology [25] and was reported according to the PRISMA-ScR [26]. We developed a protocol for this study and provide the PRISMA-ScR checklist in the Additional file 1: Appendix 1–2.

### Research questions

This scoping review seeks to answer the following two research questions:

1. What is the coverage of the four selected non-NIP vaccines (i.e., Hib, HPV, PCV and rotavirus vaccine) in China?
2. What are the barriers and challenges to improving coverage/uptake of non-NIP vaccines in China?

### Search strategy and selection criteria

We conducted a comprehensive literature search from January 1, 2013, to February 28, 2023, in five English databases (PubMed, Web of Science, EMBASE, Scopus, and WHO IRIS) and four Chinese databases (China National Knowledge Infrastructure, China Science and Technology Journal Database, Wan Fang Database, and China Biology Medicine). Our key search terms included non-NIP vaccines, Hib, HPV, PCV, rotavirus vaccine coverage, uptake, vaccination rate, challenges, barriers, and their synonyms. We used different combination sets of these key search terms for the literature search. The search strings were tailored to meet the specific requirements of each database (Additional file 1: Appendix 3).

We developed selection criteria that were mainly based on the Population, Concept, Context (PCC) framework [27]. Our study focused on the Chinese health system setting and population. We included publications in both English and Chinese, and articles meeting the following criteria were considered eligible for review: (1) Original research articles, meta-analyses, or commentaries focused on non-NIP vaccines (including the four selected vaccines) and immunization coverage in China; (2) Articles that primarily describe the barriers or challenges of uptake of non-NIP vaccines in China; (3) Articles that report at least one unfavorable factor from the perspective of government, healthcare professionals, vaccine manufacturers, and consumers of the vaccine.

Articles were excluded if they: (1) Focused on vaccine properties including efficacy, safety, and immunogenicity; (2) Only conducted cost-effectiveness, modeling, and budget impact analysis; (3) Focused on vaccines not related to the review questions; (4) Reported vaccine coverage data collected from surveys or statistics conducted in only one province or city (lack of representativeness). We further excluded certain types of publications such as clinical reports, guidelines, position reports, study protocols, book chapters, conference abstracts, editorials, duplicate studies, and studies without full text.

### Study selection and quality assessment

All retrieved literature was imported into Endnote X9 software (Clarivate, Philadelphia, USA) for screening. A group meeting was held to discuss and familiarize the research team with the eligibility criteria, and 20 randomly selected titles/abstracts were piloted to check for discrepancies. Two reviewers (MJ and XY) independently screened the titles and abstracts for relevance based on the eligibility criteria. The full-text records of the articles that met the eligibility criteria were retrieved, screened, and extracted. Any discrepancies during the screening process were resolved by third-party adjudication (SC), and consensus was reached for all decisions. We further assessed the methodological quality of all publications using the Joanna Briggs Institute Critical Appraisal Checklist according to study type [28]. Each item on the checklist for all articles was evaluated as either “present”, “not present”, “unclear” or “not applicable.” Articles without “unclear” or “not present” ratings were rated as “strong.” Articles assigned between one and three “unclear” or “not present” ratings were rated as “moderately strong” while other articles were rated as “weak.” We only included articles with strong or moderately strong quality. Critical appraisal of each included article is attached in Additional file 1: Appendix 4.

### Data extraction, charting, and analysis

A preliminary data extraction form was developed based on the research questions and piloted with 10 included articles by two independent reviewers (MJ and XY). After a research group meeting, the form was revised and finalized. For the first question, basic information (i.e., journal, publication time, authors, study settings, sample size, sampling strategy) and vaccination rates (overall and subgroup) were extracted. For the second research question, we extracted the basic study characteristics (i.e., journal, publication time, authors, research method, sampling size if applicable), main dimensions, causes of low coverage of non-NIP vaccines, relevant countermeasures, and suggestions.

The analytical process of the second research question followed the principles of thematic synthesis, and the results were structured according to Thomson’s “5A” taxonomy which organizes the possible root causes of a gap in vaccination coverage rates into five pillars (i.e., access, affordability, awareness, acceptance, and activation) [29] and is now widely applied in vaccine adoption studies [30–33]. We used the five themes to chart and analyze the findings of the second research question. We also identified sub-themes under each 5A pillar to better organize the results.

## Results

### Study characteristics

**Fig. 1:**
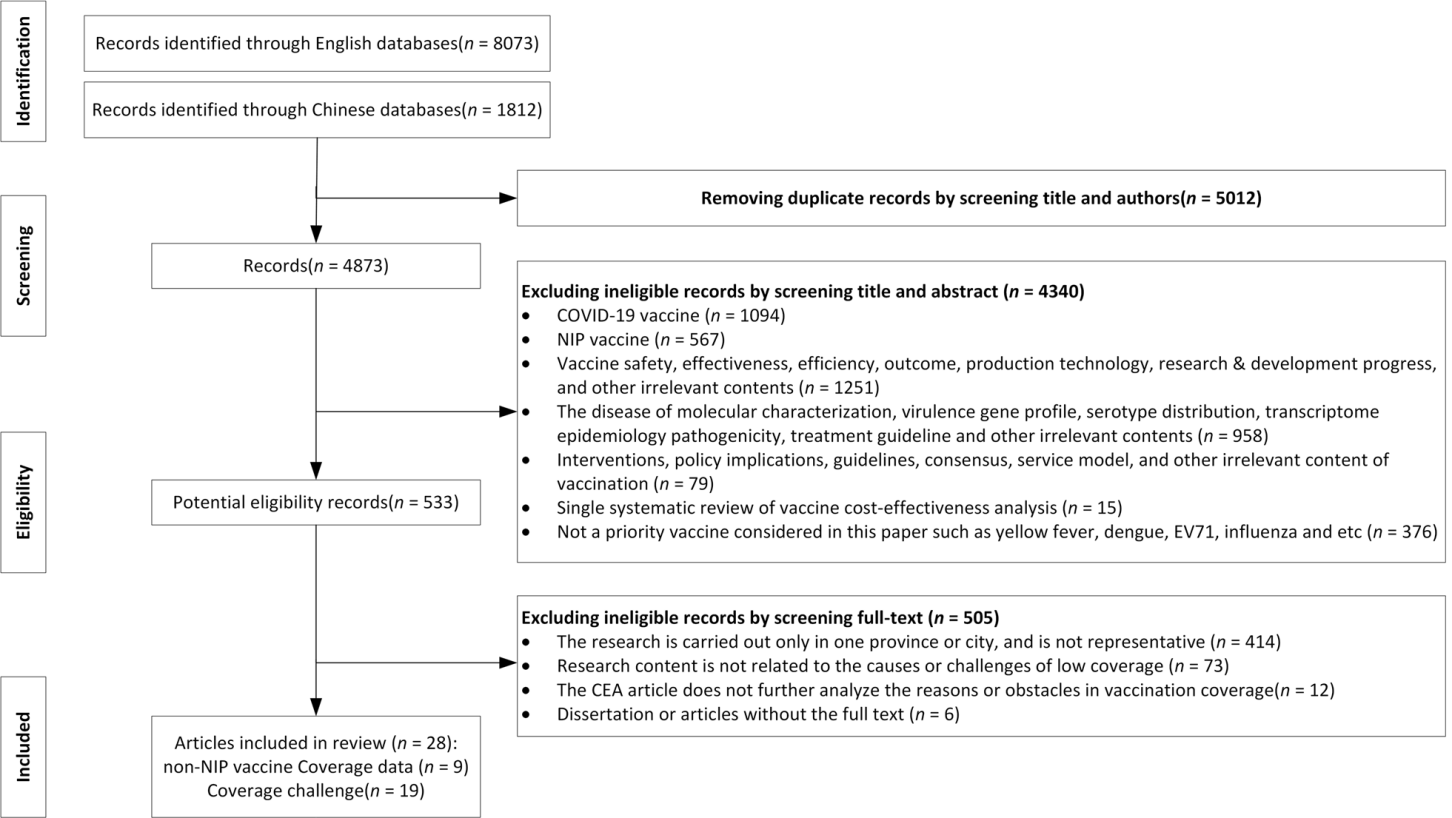
Screening and selection process

A total of 28 articles were included in this review, 9 of which focused on the coverage of the four selected vaccines and 19 on the challenges and barriers to non-NIP vaccines in China. The study selection flowchart is presented in Fig. 1.

Among the nine articles that reported vaccination rates, five articles conducted original surveys with the average number of respondents in each survey being 2904, one article utilized data from the China Immunization Information Systems (IIS), and another three articles did systematic reviews and meta-analyses. The articles generally reported a dose-specific vaccination rate, which was calculated as the number of respondents receiving the specific dose of vaccine divided by the number of total participants in the study. All nine articles reported the overall vaccination rates and eight articles compared vaccination rates across different regions. Seven articles further conducted subgroup analysis by vaccination time, dose, as well as demographic characteristics such as age, sex, and place of residency. Further details of the nine articles are presented in Table 1.

**Table 1:** Information from the nine articles about coverage of the four selected non-NIP vaccines

Vaccine	Authors and affiliations	Duration	Sample size	Methods	Study sites	Overall rate (%, 95% *CI*)	Regional disparity (%, 95% *CI*)
Hib vaccine	Li et al. [34]^b^	2006–2016	29 papers	Systematic review and meta-analysis	13 provincial-level administrative divisions (PLADs)	The pooled vaccination rate for at least one dose: 55.9, 52.3–59.4	Eastern (62.9, 58.8–67.0); central and western (48.1,40.5–55.6)
	Li et al. [35]^a^	2014	978 people	Multi-stage sampling survey	8 PLADs	44.8 (43.2–46.4) for at least one dose vaccination rate	Children in developed areas received 3 or more doses, while most children received 1 dose in underdeveloped areas
	Yang et al. [36]^b^	2007–2016	33 papers	Systematic review and meta-analysis	12 PLADs	The pooled overall coverage for at least one dose: 54.9,52.9–57.0	Eastern (59.7, 57.3–62.1); central and western (48.5,40.6–56.4)
	Lai et al. [37]^b^	2019	6668 people	Multi-stage sampling survey	10 PLADs	33.4 for three-dose at the national level in 2017	Eastern (38.1); Central (34.3); Western (26.2)
	Zhang et al. [38]^b^	2019	5294 people		10 PLADs	Hib1(42.6, 41.3–44.0), Hib3 (25.0, 23.7–26.3)	The coverage in developed areas is generally higher than that in underdeveloped areas in 10 provinces
HPV vaccine	Song et al. [39]^a^	2018–2020	NA	Analysis of routine statistics data	31 PLADs	The estimated three-dose cumulative coverage in the year 2018, 2019, 2020 (0.3, 1.0, 2.2)	Beijing (8.3), Shanghai (7.4), Xinjiang (0.5), Qinghai (0.4), Tibet (0.1) in 2020
PCV vaccine	Yue et al. [40]^a^	2014	978 people	Multi-stage sampling survey	8 PLADs	16.9 for one dose of PPSV23 vaccination	N/A
	Shao et al. [41]^b^	2013–2021	76 papers	Systematic review and meta-analysis	NA	The summary PCV vaccine coverage: 21.7,17.2–26.5	Eastern (22.8, 17.7–28.5) Central (22.1, 8.5–35.4) Western (19.8, 9.0–37.6)
	Lai et al. [37]^b^	2019	6668 people	Multi-stage sampling survey	10 PLADs	1.3 for three-dose at the national level in 2017	Eastern (2.5); Central (0.6); Western (0.7)
	Zhang et al. [38]^b^	2019	5294 people		10 PLADs	PCV1(7.7,6.9–8.4), PCV3(5.1, 4.5–5.8)	The coverage in developed areas is generally higher than that in underdeveloped areas in 10 provinces
Rotavirus vaccine	Liu et al. [42]^a^	2014	606 people	Multi-stage sampling survey	6 PLADs	Rota1, Rota2, Rota3 (32.8, 9.7, 3.5)	One dose of rotavirus vaccine in high, middle, and low-income areas (45.0, 37.7, 15.5)
	Zhang et al. [38]^b^	2019	5294 people		10 PLADs	Rota1 (20.3, 19.2–21.3) Rota3 (1.8, 1.3–2.2)	The coverage in developed areas is generally higher than that in underdeveloped areas in 10 provinces

^a^First author affiliation: China CDC

^b^First author affiliation: University. *NIP* National Immunization Program; *CI* Confidence interval; *Hib* Haemophilus influenzae type b; *HPV* Human papillomavirus; *PLADs* Provincial-level administrative divisions; *PCV* Pneumococcal conjugate vaccine; *PPSV* Pneumococcal polysaccharide vaccine; *NA* not applicable

Fifteen of the nineteen articles discussing the challenges of increasing non-NIP vaccine coverage were published in 2020 or later, and were contributed to by local and national Centers for Disease Control and Prevention (CDCs) and universities. Thirteen of these articles were original articles using quantitative (*n* = 7), qualitative (*n* = 2), mixed and review methods (*n* = 4), and another six were commentaries (Details of the nineteen articles are in Table 2).

**Table 2:** Information from the nineteen included articles about non-NIP vaccine coverage barriers

Authors and affiliations	Publication year	Methods and vaccine type	Study sites	Sample size	Access				Affordability		Awareness	Acceptance				Activation
					Capacity of the service provider	Ease of access to services	Place of residency	Supply of vaccines	Vaccine price	Government subsidy	Vaccine awareness	Vaccine safety and efficacy	Perceived susceptibility of being infected	Personal health beliefs	Interpersonal and media influence	Effective interventions
Hou et al. [43]^c^	2014	Stratified sampling survey ^e^	3 provinces	1924 respondents					**√**	**√**	**√**	**√**	**√**	**√**	**√**	
Chang et al. [44]^c^	2019	Multistage stratified random sampling survey ^d^	3 provinces	1791 households	**√**	**√**	**√**		**√**			**√**			**√**	
Deng et al. [45]^c^	2021	Convenience sampling survey^f^	4 PLADs	1022 participants					**√**		**√**	**√**	**√**		**√**	
Wang et al. [46]^c^	2021	Proportional sampling survey ^d^	31 PLADs	7318 respondents							**√**	**√**	**√**	**√**	**√**	
Si et al. [47]^c^	2021	Stratified sampling survey ^f^	7 PLADs	3867 students				**√**	**√**		**√**	**√**	**√**	**√**	**√**	
Lai et al. [48]^c^	2022	Multi-stage stratified sampling ^e^	10 PLADs	1138 healthcare workers, 2973 older adults aged ≥ 65							**√**	**√**		**√**	**√**	
Yin et al. [49]^c^	2023	Multi-stage sampling survey ^f^	31 PLADs	5959 respondents			**√**		**√**		**√**	**√**	**√**	**√**	**√**	
Duan et al. [50]^a^	2016	Commentary^d^					**√**		**√**		**√**	**√**		**√**	**√**	
Zhang et al. [51]^b^	2018	Commentary^d^				**√**	**√**	**√**	**√**	**√**	**√**	**√**	**√**	**√**	**√**	
Wang et al. [52]^a^	2020	Commentary^d^				**√**	**√**	**√**	**√**	**√**	**√**	**√**	**√**	**√**	**√**	
Wang et al. [53]^c^	2020	Commentary^d^							**√**		**√**	**√**				**√**
Wang et al. [54]^a^	2021	Commentary^f^			**√**			**√**	**√**		**√**	**√**	**√**		**√**	
Wang et al. [55]^b^	2022	Commentary^d^			**√**	**√**		**√**		**√**	**√**	**√**			**√**	
Wang et al. [56]^c^	2020	Systematic review^d^		58 papers							**√**	**√**	**√**	**√**	**√**	
Bai et al. [57]^b^	2022	Narrative review^e^					**√**		**√**		**√**	**√**	**√**		**√**	
Wang et al. [58]^c^	2022	Systematic review^f^		73 papers					**√**		**√**	**√**	**√**			
Gong et al. [59]^c^	2021	Qualitative interview^d^	3 provinces	26 vaccination providers&160 caregivers	**√**	**√**										
Lin et al. [60]^c^	2022	Qualitative interview^d^	3 provinces	26 interviewees	**√**		**√**	**√**	**√**		**√**	**√**			**√**	**√**
Han et al. [61]^c^	2022	Multi-stage sampling survey and qualitative interview^d^	3 provinces	555 for the survey and 49 for the interview	**√**			**√**	**√**							

*NIP* National Immunization Program; *PLADs* Provincial-level administrative divisions; *PCV* Pneumococcal conjugate vaccine; *NA* not applicable

^a^First author affiliation: China CDC

^b^First author affiliation: Provincial and municipal CDC

^c^First author affiliation: University

^d^Vaccine type: non-NIP vaccine

^e^Vaccine type: PCV vaccine

^f^Vaccine type: HPV vaccine

### Current status of non-NIP vaccine coverage in China

Five articles reported Hib vaccination rates. Within three articles, the pooled vaccination rate for at least one dose ranged from 44.8% to 55.9% [34–36]. Additionally, the three-dose Hib vaccination rate ranged from 25.0% to 33.4% in two surveys [37, 38].

We identified only one article that reported coverage of the HPV vaccine in China using data from IIS. The estimated three-dose coverage was 2.2% among women aged 9 to 45 years old in 2020 [39].

Four articles showed coverage rates of the PCV vaccine. According to one systematic review, the pooled vaccination rate was 21.7% (95% *CI*: 17.2–26.5%) [41]. The other three surveys reported a three-dose rate ranging from 1.3% in 2017 to 5.1% [37, 38] and PPSV-23 coverage of 16.9% in 2014 [40].

Two articles reported rotavirus vaccine uptake rates. the first-dose vaccination rate was reported to be 20.3% in 2014 [42] and 20.3% in 2019 [38].

These findings suggest significant regional disparities in vaccine coverage. In 2017, the three-dose PCV vaccination rate in eastern provinces was 2.5%, whereas in central and western provinces it was only 0.6% and 0.7%, respectively. In the same year, 75.8% of children under five in Shanghai received the full Hib vaccination, while in areas with a greater burden of the disease, such as Xinjiang, less than 3.0% of children were fully vaccinated. A similar situation was also observed for rotavirus vaccines [37]. The one-dose vaccination rate of rotavirus vaccines in Shanghai was reported to be 47.0%, which was only 8.4% in Gansu Province, located in the western region [38]. The cumulative estimated rate of HPV vaccine for women aged 9–45 in 2020 showed that Beijing and Shanghai reached 8.3% and 7.4%, respectively, while Tibet, Qinghai, and Xinjiang only reached 0.1%, 0.4%, and 0.5%, respectively [39]. Table 1 provides a comprehensive description of the detailed information regarding vaccination rates.

### Barriers and challenges in increasing coverage of non-NIP vaccines

We further identified 12 sub-themes within the 5A framework and reported the frequency of each theme being mentioned by the nineteen articles (Table 3). Within the 5A framework, acceptance, awareness, and affordability stood out as the most prominent themes. The most common acceptance factors were related to perceived vaccine safety, vaccine efficacy, and interpersonal and media influence. The affordability factors that were most mentioned were related to vaccine price.

**Table 3:** Theme and sub-themes distribution of non-NIP vaccine coverage challenges

Thematic dimension	Frequency (percentage)
Access [44, 47, 49–52, 54, 55, 57, 59–61]	12 (63.2%)
Capacity of the service provider [44, 54, 55, 59–61]	6 (31.6%)
Ease of access to services [44, 51, 52, 55, 59]	5 (26.3%)
Place of residency [44, 49–52, 57, 60]	7 (36.8%)
Supply of vaccines [47, 51, 52, 54, 55, 60, 61]	7 (36.8%)
Affordability [43–45, 47, 49–55, 57, 58, 60, 61]	15 (78.9%)
Vaccine price [43–45, 47, 49–54, 57, 58, 60, 61]	14 (73.7%)
Government subsidy [43, 51, 52, 55]	4 (21.1%)
Awareness [43, 45–58, 60]	16 (84.2%)
Vaccine awareness [43, 45–58, 60]	16 (84.2%)
Acceptance [43–58, 60]	17 (89.5%)
Vaccine safety and efficacy [43–58, 60]	17 (89.5%)
Perceived susceptibility of being infected [43, 45–47, 49, 51, 52, 54, 56–58]	11 (57.9%)
Personal health beliefs [43, 46–52, 56]	9 (47.4%)
Interpersonal and media influence [43–52, 54–57, 60]	15 (78.9%)
Activation [53, 60]	2 (10.5%)
Effective interventions [53, 60]	2 (10.5%)

“Frequency” referred to the number of times a particular theme appears in each article, and the corresponding “percentage” was calculated by dividing the number of occurrences by the total number of 19 articles

*NIP* National Immunization Program

## Access

Challenges associated with vaccine access included the capacity of immunization service providers, the ease of accessibility to vaccination services, and the location of residence, all of which exert an impact on individuals’ ability to reach the vaccine and its services. This theme was mentioned in 12 articles, and we identified four sub-themes related to it.

### Capacity of the service provider

Six articles discussed service provider capacity. The capacity of the service provider refers to both having a sufficient number of vaccinators and having vaccinators with professional public health training and vaccination licenses. In areas with a high volume of vaccination services, the staff could not meet the demand in the chain of services including vaccination appointments, consultation, registration, and follow-up [54]. The staff always had to take on additional public health responsibilities besides providing vaccination services, such as chronic disease management and health check-ups for the elderly. Additionally, a substantial proportion of young staff were temporary workers, encountering formidable obstacles in attaining permanent employment status, thus leading to their subsequent resignations and creating a shortage of human resources. Further, the lack of a unified electronic information system also increased staff workload [59, 61].

Besides the heavy workload, the perceived risks of providing non-NIP vaccination services and the lack of financial incentives further constrained capacities. The risks came from possible adverse reactions after vaccination [61], and the absence of standard vaccination guidelines, especially for the elderly [55]. In addition, vaccinators were not enthusiastic about recommending non-NIP vaccines due to insufficient financial incentives and performance review requirements [61].

### Ease of access to services

Five articles discussed access to vaccination services. These articles referred to whether vaccination services were provided at a time and place that are easily accessed, which could be an important factor in vaccination decision-making, especially for those who live in remote areas. Ease of access to services was determined by key factors including the locations of the service providers and users, the density of vaccination facilities, vaccination service provision time (weekdays or weekends), space of vaccination clinics, and transportation cost [44, 51, 52, 55, 59]. In comparison, residents perceive vaccination services more favorably when there is improved transportation convenience, centralized and weekend-focused opening hours, and better facilities and environments for vaccination institutions.

### Place of residency

Seven articles evaluated place of residency as a barrier to vaccination. The vaccination rates among rural-to-urban migrants, children that were left behind, and rural residents were reported to be lower when compared with the urban or permanent residents, particularly among the elderly and children [44, 50–52, 57]. Left-behind children were usually raised by grandparents whose vaccination coverage was approximately 5–15% lower than non-left-behind/host children. On the one hand, vaccination reminders were normally sent to parents who may be far away or unable to communicate regularly, resulting in a delay in receiving the necessary information. On the other hand, grandparents tended to be less educated and had limited knowledge of vaccines compared to parents [60]. Additionally, ethnic minority children (e.g., Yi, Zang, Qiang) had lower vaccination rates due to language barriers and religious beliefs, which made it difficult to convey the importance of vaccination to parents [60].

### Supply of vaccines

Seven articles discussed an adequate vaccine supply as a prerequisite for effective and timely vaccination. Less commonly used vaccines may only be procured monthly or quarterly, leading to insufficient vaccine supply and causing people to forgo vaccination [52]. Some vaccines such as the HPV vaccine are not yet available in many underdeveloped or rural areas [47]. Unexpected events, such as market changes or vaccine shortages, also resulted in vaccine supply issues. For instance, vaccine shortages occurred intermittently after the provision of vaccines was deemed to be substandard by a biotechnology company in 2018 [60]. After the COVID-19 outbreak, the high demand for PCV vaccines outpaced the available supply, primarily due to the lengthy processing time required for batch approval, production, and distribution of vaccines [57].

## Affordability

Barriers to affordability were related primarily to high vaccine prices, given the low income level of the households concerned, and the extent to which government subsidies are offered. This topic was covered in 15 of 19 included articles.

### Vaccine price

Fourteen articles demonstrated that the current price of non-NIP vaccines in China was generally too expensive for the average family. For instance, the cost of the non-NIP vaccination scheme in 2019 of Hebei Province for boys from birth to 6 years was 7122 Chinese Yuan (CNY), equivalent to around 988 United States Dollars (USD, exchange rate = 1:7.2) for eight types of recommended vaccines, while girls needed to pay an additional 1160 CNY (around 161 USD) for the bivalent HPV vaccine [52]. Completing the full vaccination schedule as recommended imposed a significant financial burden on caregivers. Furthermore, there is a price discrepancy between domestically produced and imported non-NIP vaccines. In China, the price of imported HPV vaccine ranges between 1806 and 4000 CNY (around 251–556 USD), while the domestic HPV vaccine is priced at 658–987 CNY (around 91–137 USD) [58].

### Government subsidy

Four articles showed that government subsidies could alleviate the financial burden of vaccination and increase vaccine uptake [43, 51]. For example, Shanghai implemented a policy that has allowed residents aged 60 years or older to receive free PPSV-23 vaccines since 2013. This initiative has resulted in a substantial increase in vaccine coverage, with a total of 1.56 million people benefiting from the program [57]. In areas where vaccination was reimbursed or provided freely, the vaccination rate among the elderly was much higher than that of the areas without corresponding policies [55].

## Awareness

Sixteen articles covering awareness were identified and barriers were related to individuals’ ability to acquire information about the necessity, benefits, and potential risks associated with recommended vaccines.

### Vaccine awareness

Having correct and sufficient vaccine knowledge was found to increase people’s willingness to pay for non-NIP vaccines and facilitate their vaccination decisions [43]. However, a lack of vaccine knowledge could lead to uncertainty in estimating the benefits of vaccination. For example, data from a survey indicated that individuals with a higher level of knowledge concerning pneumonia were 1.39 times more likely to receive the PCV compared to those with limited knowledge [57]. One article mentioned that only 12.9% of adolescents were aware of HPV and its related diseases, and less than one-third of adults had heard of the HPV vaccine [54].

## Acceptance

The primary barriers to acceptance were concerns about vaccine safety and efficacy, as well as a reliance on perceived susceptibility to infection and health beliefs. This was the most discussed topic (17 articles mentioned it) and was further grouped into the four sub-themes below.

### Vaccine safety and efficacy

Vaccine safety and efficacy were the most common concerns influencing decision-making. Specifically, the protection effect and duration, potential adverse effects of vaccination, and the quality and safety of the vaccine were all important factors that affected an individual’s decision to get vaccinated. Less than 50% of the elderly population in a survey expressed the belief that vaccination could provide protection against pneumococcal disease and lacked reasonable perceptions about vaccination [57]. College students surveyed also expressed uncertainty about getting the HPV vaccine, expressing concerns about safety and effectiveness as the main reasons [45]. In some areas of China, vaccine safety incidents hindered access to reliable vaccine information, emphasizing the need for transparent and evidence-based communication from health officials to address public concerns and customize messages for individuals with different educational backgrounds [58]. People tended to prefer non-NIP vaccines that covered more diseases. For example, the administration of 9- and 4-valent HPV vaccines was significantly greater than that of 2-valent HPV vaccines, while the 23-valent pneumococcal conjugate vaccine demonstrated a higher frequency of dosing compared to the 13-valent pneumococcal conjugate vaccine [50–52]. Combination vaccines may therefore be a solution that increases uptake by reducing the burden of multiple doses.

### Perceived susceptibility of being infected

The uncertainty of disease occurrence posed challenges for individuals trying to assess the potential risk of being infected. In this regard, the perceived severity of the disease and the judgment of one’s own physical condition played a crucial role in an individual’s risk assessment. For instance, when parents decided whether or not to vaccinate their child, they considered the actual risk of disease occurrence and the perceived negative health outcomes associated with the disease. The desire to avoid negative health outcomes and the expectation of potential health risks contributed to an individual’s motivation for vaccination. Thus, individuals may generally prefer vaccination due to the higher perceived risk of disease and negative health outcomes [52, 56, 58].

### Personal health beliefs

Non-NIP vaccination was a highly autonomous and selective process, whereby those with positive health beliefs might actively seek information about non-NIP vaccines and consult professionals about making an appointment for vaccination [47]. However, personal health beliefs were complex and could be influenced by several factors, such as cultural background, occupation, personal and family income, vaccine-preventable disease experience, and positive vaccination experience. These factors have been identified as strong predictors of vaccination behavior [43, 47, 49, 50].

### Interpersonal and media influence

People obtained information about non-NIP vaccines through various sources, including healthcare workers, social media, and peers. Healthcare providers with better knowledge and attitudes toward non-NIP vaccines could positively influence the decision-making of getting vaccinated. Peer influence from family, friends, and communities such as pregnant women also played an important role, especially for those with limited knowledge about the disease and vaccination [43]. Public health workers were more likely to recommend non-NIP vaccines than general practitioners, possibly due to differences in medical education [48]. A survey showed that the decision to receive a non-EPI vaccine was influenced by various sources of information, with doctors accounting for 66.3% of the respondents, followed by family or friends at 55.8%, and social media at 30.1% [46].

The internet and new media have become increasingly influential in shaping public opinion about non-NIP vaccines in China. The unbiased dissemination of scientific evidence and facts could effectively promote vaccination efforts by increasing people’s knowledge about vaccines. However, negative events, opinion baiting, and media sensationalism might trigger public opinion crises, leading to a significant drop in vaccination rates [51].

## Activation

The impediments to participation in vaccination programs were linked to whether using strategies for prompting or incentivizing individuals who expressed an intention to receive the vaccine to take proactive measures.

### Effective interventions

Two articles discussed activation and mentioned several measures that had proven effective in boosting vaccination rates including utilizing routine maternal and child healthcare visits for catch-up vaccination and conducting follow-up visits to all families assisted by village doctors [60]. Some schools encouraged students to receive vaccinations, such as PCV vaccines, to prevent campus infections during autumn and winter when respiratory diseases were more prevalent. Non-mandatory vaccination policies such as awareness campaigns, mobile vaccination vans, and education and training on vaccine-preventable diseases were also in place in hospitals [53].

## Discussion

This review systematically summarizes the coverage of four non-NIP vaccines and the challenges to increasing their uptake in China. As a part of the national immunization strategy, non-NIP vaccines have also played a significant role in improving population immunity and reducing the burden of vaccine-preventable diseases. Our scoping review focused on the four routine vaccines recommended by the WHO to include in the immunization programs of all Member States, which are Hib, HPV, PCV, and rotavirus vaccines. Our scoping review of 28 documents provides robust quantitative evidence suggesting relatively low coverage of the non-NIP vaccines and identifying key barriers to increasing coverage that are grouped under the 5A framework: Access, Awareness, Affordability, Acceptance, and Activation.

The coverage rates of Hib, HPV, PCV, and rotavirus vaccines in China are significantly lower than the global average, the Global Vaccine Alliance (GAVI)-eligible low-income countries, and other low-and-middle-income countries (LMICs). China is the only country that has not included Hib in its NIP, and the only country that has not included PCV in its NIP among the East Asian countries, except North Korea. Support from GAVI has helped boost the uptake of these four vaccines in eligible low-income countries and the coverage for these four vaccines is generally above 80% [62]. LMICs such as India and South Africa have a coverage of over 70% [62]. However, in the case of China, these four vaccines have not been included in the NIP and the coverage rates are low, which can be attributed to several key barriers, including high vaccine prices, insufficient vaccine awareness, and concerns among the general public about vaccine safety and efficacy.

The coverage of four selected non-NIP vaccines was much lower than NIP vaccines in China. In addition, we found that the coverage of the four vaccines was even lower in less developed regions of China. Among the four vaccines, coverage for at least one dose of the Hib vaccine was the highest (54.9–55.9% from two meta-analyses) in 2016 [34, 36] while coverage of the other three vaccines was lower than 30%. Notably, coverage of the four vaccines in China is far lower than the world average and many developing countries. For example, the full-dose vaccination rate of Hib, PCV, and rotavirus vaccines was 71%, 51%, and 49% globally, respectively, and 21% of girls by age 15 around the globe received at least one dose of HPV vaccine in 2021[62]. Unequal access to non-NIP vaccines across regions could cause an avoidable burden of VPDs and exacerbate regional healthy inequity.

We identified 12 factors that affect the coverage of selected non-NIP vaccines under the 5A framework. Among the identified barriers, high price, low vaccine awareness, and concerns about vaccine safety were mentioned most in the articles we included. We further examined and analyzed these barriers from both the supply and demand perspectives, which permits a comprehensive understanding of the multifaceted aspects that affect vaccination coverage and allows for the discovery of potential solutions to address the challenges effectively.

On the supply side, the main challenges of low uptake include high prices, low production, and insufficient incentives for vaccinators. The overall price of non-NIP vaccines is high in China and far exceeds the average price across the globe. The main reasons for the high prices in China include the single financing channel, lack of centralized bidding and procurement, and high marketing costs for vaccine manufacturers. The low production of non-NIP vaccines in China is largely due to the unpredictability of the demand in the domestic market. Currently, county-level CDCs report the procurement needs to provincial CDCs based on historical vaccination data, and vaccine manufacturers cannot fully respond to the sudden increase or decrease in demand. There is no regular non-NIP information system to support the demand estimation. In addition, factors such as declining birth rates, stricter national vaccine approval and issuance regulations, and the shelf life of vaccines make it more difficult for manufacturers to predict market demand. In addition, there is no incentive for healthcare workers to provide non-NIP vaccination services. No markup has been allowed for non-NIP vaccines since 2016 and only very low (around 20 CNY = 2.77 USD) vaccination service fees can be charged according to the Immunization Administration Law [63].

On the demand side, key factors that affect vaccination rates include family income, caregiver education, vaccine awareness, and vaccine hesitancy. First, the general public has an inadequate understanding of the role of non-NIP vaccines. Most people lack sufficient awareness of non-NIP vaccines, especially in economically average or underdeveloped areas. Immunization institutions (including doctors, nurses, and other medical personnel) and the mass media have not provided sufficient and quality education on non-NIP vaccines. Second, vaccination rates for non-NIP vaccines among children from high-income families are significantly higher due to the high vaccine price. There is a substantial income gap across regions in China. In 2021, the per capita disposable income was 35,128 CNY (around 4881 USD), which was 1.6 times higher in eastern region than western region, and 2.5 times higher in urban areas than rural areas [64]. Third, caregiver education levels, especially the mother's education level, plays an important role in determining a child's vaccine uptake. However, many children are taken care of by grandparents who may not have sufficient knowledge about the importance of vaccines. Finally, the occurrence of vaccine safety incidents, leading to a lack of trust, has also to some extent exacerbated vaccine hesitancy and weakened vaccination willingness.

Including these key non-NIP vaccines in the NIP program could address most of the barriers identified in the review. The key features and advantages of including a vaccine in the NIP include: (1) It is a government recommendation for the vaccine, which means that the government believes the vaccine to be important for all children and is obliged to provide the vaccine; (2) The use of the vaccine is promoted as an important health product for children; (3) The vaccine is provided at no cost to the family; (4) There are technical recommendations for proper use of the vaccine, which non-NIP vaccines lack; (5) Schools are required to assess the coverage of the vaccine and refer children in need of the vaccine to clinics; (6) The vaccine will be included in the government’s vaccine injury compensation program; (7) The family shares the duty with the government to vaccinate the child. Considering the significant and sustained financial and human resources required to integrate these vaccines into the program, the government's prudent approach would be to incorporate them step by step gradually. To achieve this goal, the proposed strategy involves prioritizing vaccines based on key factors such as disease burden, financial resources, and market readiness. Special attention should be given to high-risk populations, areas with a severe disease burden, and underdeveloped regions, ensuring their inclusion in the national immunization plan before further expansion. This approach emphasizes the need for careful planning as well as a systematic approach to achieving sustainable success.

We also propose several additional options to help China achieve relevant goals set in the IA2030, SDG, and Healthy China 2030 and to ensure everybody has equal access to life-saving vaccines. First, it is essential to generate high-quality evidence on the vaccination rate of key non-NIP vaccines and the disease and economic burden of relevant VPDs. One important message from our scoping review was the severe lack of high-quality and updated data on the coverage of non-NIP vaccines in China. Available data could not support generation of a pooled rate given that the articles included had different study timeframes, sample populations, and methods. Second, improve the financing, bidding, and procurement of non-NIP vaccines. In addition to out-of-pocket payments, other financing channels such as health insurance funds, commercial health insurance, and local fiscal funding could be explored to cover the cost of the vaccines. Regarding bidding and procurement, it is helpful to draw on experiences of centralized bidding and procurement for drugs and international good practices on vaccine bidding and procurement for optimization. Third, increase the incentives for vaccinators to stimulate their initiatives to recommend critical non-NIP vaccines. Fourth, strengthen health education on vaccines to increase public awareness and reduce vaccine hesitancy.

The current study has several strengths and limitations. The first strength is that we did an extensive review of the literature available on both the coverage of the selected non-NIP vaccines and the barriers and challenges behind the low uptake. Second, we adopted a relatively long timeframe, i.e., ten years, to collect the evidence available. Third, we included both English and Chinese literature in this scoping review to facilitate a more comprehensive understanding of this topic for the international community. Regarding limitations, first, we did not generate a pooled estimate of the coverage for the selected vaccines due to inconsistency in the methods and different dose-specific vaccine rates calculated in the studies. Second, our review did not include relevant articles published before 2013 due to time relevance. Third, our review did not include any grey literature which may also contain rich information about the research topics.

## Conclusions

The Hib, HPV, PCV and rotavirus vaccines are not included in China’s NIP and their coverage is much lower than the world average. In addition, their uptake is even lower in less developed regions of China. High vaccine prices, insufficient vaccine awareness, and concerns about vaccine safety and efficacy are the main barriers to increasing uptake. Concerted efforts from the government, the public, and society are required to tackle the barriers and challenges identified in this study, both on the demand and supply side, to ensure everybody has equal access to life-saving vaccines in China. Particularly, the government should take a prudent approach to gradually incorporate these four vaccines into the NIP step by step, and make a prioritizing strategy based on key factors such as disease burden, financial resources, and market readiness, with special attention to high-risk populations and underdeveloped regions.

### Supplementary Information

**Additional file 1**: **Appendix 1: **The coverage and challenges of increasing uptake of non-National Immunization Program vaccines in China - protocol . **Appendix 2: **Preferred Reporting Items for Systematic reviews and Meta-Analyses extension for Scoping Reviews (PRISMA-ScR) Checklist. **Appendix 3: **Search Strategy and Results. **Appendix 4: **JBI Critical Appraisal Checklist.

## Data Availability

The sources of case information were listed in the Additional file 1: Appendix. All data and materials used are available from the corresponding author on reasonable request.

## Abbreviations

*CI*: Confidence interval
CDC: Centers for Disease Control and Prevention
CNY: Chinese Yuan
GAVI: The Global Vaccine Alliance
Hib: Haemophilus influenzae type b
HPV: Human papillomavirus
IA2030: Immunization Agenda 2030
IIS: Immunization Information Systems
LMICs: Low-and-middle-income countries
NIP: National Immunization Program
Non-NIP: Non-National Immunization Program
PCV: Pneumococcal conjugate vaccine
PLADs: Provincial-level administrative divisions
PPSV: Pneumococcal polysaccharide vaccine
PRISMA-ScR: Preferred Reporting Items for Systematic Reviews and Meta-Analyses Extension for Scoping Reviews
SDGs: Sustainable Development Goals
USD: United States Dollars
VPDs: Vaccine-preventable diseases
WHO: World Health Organization
