# Supplementary material for: The coverage and challenges of increasing uptake of non-National Immunization Program vaccines in China: a scoping review
Source: Infect Dis Poverty. 2023 Dec 8;12:114. doi: 10.1186/s40249-023-01150-8 (PMC10704715; doi:10.1186/s40249-023-01150-8)
Supplement: Supplementary file 1 — Additional file 1: Appendix 1: The coverage and challenges of increasing uptake of non-National Immunization Program vaccines in China - protocol . Appendix 2: Preferred Reporting Items for Systematic reviews and Meta-Analyses extension for Scoping Reviews (PRISMA-ScR) Checklist. Appendix 3: Search Strategy and Results. Appendix 4: JBI Critical Appraisal Checklist. [file 40249_2023_1150_MOESM1_ESM.docx]

**Appendix**

**Contents**

**[Appendix 1](#_Toc134143929)**

[The coverage and challenges of increasing uptake of non-National Immunization Program vaccines in China - protocol 1](#_Toc134143930)

**[Appendix 2](#_Toc134143931)**

[Preferred Reporting Items for Systematic reviews and Meta-Analyses extension for Scoping Reviews (PRISMA-ScR) Checklist 3](#_Toc134143932)

**[Appendix 3](#_Toc134143933)**

[Search Strategy and Results 5](#_Toc134143934)

**[Appendix 4](#_Toc134143935)**

[JBI Critical Appraisal Checklist 16](#_Toc134143936)

# **Appendix 1**

# **The coverage and challenges of increasing uptake of non-National Immunization Program vaccines in China: a scoping review - protocol**

**Background**

International studies have shown significant differences in vaccine uptake by age, gender, ethnicity, geography, and socioeconomic status. In China, there are similar inequities and distribution differences in the vaccination of non-National Immunization Program (non-NIP) vaccines, making it difficult for non-NIP vaccines to achieve optimal coverage.

Evidence relevant to non-NIP vaccine coverage and problems is lacking, particularly given the need for public health vaccine uptake in western regions where resources are limited.

To identify barriers to vaccine coverage improvements, we plan to conduct a scoping review and categorize the issues. The results of the scoping review will guide the development of vaccine uptake promotion tools and policy implementation.

**Central question**

What is the current status of the literature on non-NIP vaccination coverage especially for key vaccines? Are there any obstacles to non-NIP vaccine coverage in China?

**Review objectives**

The objectives of the review are to (a) describe how vaccination rates in non-NIP vaccines are characterized in the academic literature, mainly four priority vaccines including PCV, Hib, HPV, and rotavirus; (b) describe major problems and roots in non-NIP vaccination coverage, and (c) provide an overview of the gaps and future recommendations for research.

**Review Methods**

Justification

The proposed paper will be a scoping review. Scoping reviews are appropriate “to determine the scope or coverage of a body of literature on a given topic and give a clear indication of the volume of literature and studies available as well as an overview (broad or detailed) of its focus. Scoping reviews are useful for examining emerging evidence when it is still unclear what other, more specific questions can be posed and valuably addressed by a more precise systematic review”.

Inclusion and exclusion criteria

-Population: This study focuses on Chinese, with no limitations on age, sex, ethnicity, etc.

-Concept: Non-NIP Vaccine in China refers to voluntary and self-paid vaccines. China now currently has more than 30 types of non-NIP vaccines, noteworthy, some of the non-NIP vaccines in China have been included in the national immunization program in other countries, but the four vaccines focused on in this study (HPV, rotavirus, PCV, and Hib vaccine) are currently available in China at one's own expense. The research should describe the barriers or challenges to the uptake of non-NIP vaccines in China and this is the most important review criterion. Studies will be excluded those focus on the vaccine properties (efficacy, safety, immunogenicity, etc.) and are cost-effectiveness, modeling, and budget impact analysis.

-Context: All the studies are in China’s healthcare setting.

-Research detail:

a) Language: English and Chinese;

b) Published Time:2013-Feb.2023;

c) Study method: no limitation;

d) Type: clinical reports, guidelines, position reports, book chapters, conference abstracts, editorials, duplicate studies, and studies without full text and peer-reviewed will be excluded.

Search strategy

-Database

a) Chinese database: China National Knowledge Infrastructure, China Science and Technology Journal Database, Wan Fang Database, China Biology Medicine;

b) English database: PubMed, Web of Science, EMBASE, Scopus, WHO IRIS.

-Search keyword

a) Non-immunization program immunization vaccine/NIP vaccine, category/class two (2) vaccine, self-paid vaccine, optional vaccine, out-of-pocket vaccine, non-EPI vaccine;

b) Hapillomaviridae/human papilloma virus/HPV, Pneumonia/Pneumococcal/Pneumovax, Rotavirus/RotaTeq, Haemophilus, influenzae type b/Hib;

c) Coverage, uptake, vaccination rate;

d) Challenge, barrier, problem, issue, reason, roots, determinants.

-Search steps

Based on inclusion and exclusion criteria, the reviewers screen all titles and abstracts of retrieved citations for inclusion. Two reviewers will independently evaluate the full text of articles potentially meeting eligibility criteria. A discussion will be held to resolve any discrepancies. Where a consensus cannot be reached, a third reviewer will arbitrate.

**Data extraction**

We will conduct the review according to PRISMA (Preferred Reporting Items for Systematic Reviews and Meta-Analyses) guidelines. Data from included reviews will be extracted by one reviewer and independently checked by a second reviewer. We will extract the basic information of included studies and the key issues, the data extraction table will be provided later.

# Appendix 2

# Preferred Reporting Items for Systematic reviews and Meta-Analyses extension for Scoping Reviews (PRISMA-ScR) Checklist

| SECTION | ITEM | PRISMA-ScR CHECKLIST ITEM | REPORTED ON PAGE # |
| --- | --- | --- | --- |
| **TITLE** | | | |
| Title | 1 | Identify the report as a scoping review. | 1 |
| **ABSTRACT** | | | |
| Structured summary | 2 | Provide a structured summary that includes (as applicable): background, objectives, eligibility criteria, sources of evidence, charting methods, results, and conclusions that relate to the review questions and objectives. | 2 |
| **INTRODUCTION** | | | |
| Rationale | 3 | Describe the rationale for the review in the context of what is already known. Explain why the review questions/objectives lend themselves to a scoping review approach. | 3-4 |
| Objectives | 4 | Provide an explicit statement of the questions and objectives being addressed with reference to their key elements (e.g., population or participants, concepts, and context) or other relevant key elements used to conceptualize the review questions and/or objectives. | 4 |
| **METHODS** | | | |
| Protocol and registration | 5 | Indicate whether a review protocol exists; state if and where it can be accessed (e.g., a Web address); and if available, provide registration information, including the registration number. | 4 and appendix1 |
| Eligibility criteria | 6 | Specify characteristics of the sources of evidence used as eligibility criteria (e.g., years considered, language, and publication status), and provide a rationale. | 4 |
| Information sources* | 7 | Describe all information sources in the search (e.g., databases with dates of coverage and contact with authors to identify additional sources), as well as the date the most recent search was executed. | 4 and appendix 3 |
| Search | 8 | Present the full electronic search strategy for at least 1 database, including any limits used, such that it could be repeated. | 4 and appendix 3 |
| Selection of sources of evidence† | 9 | State the process for selecting sources of evidence (i.e., screening and eligibility) included in the scoping review. | 5 |
| Data charting process‡ | 10 | Describe the methods of charting data from the included sources of evidence (e.g., calibrated forms or forms that have been tested by the team before their use, and whether data charting was done independently or in duplicate) and any processes for obtaining and confirming data from investigators. | 5 |
| Data items | 11 | List and define all variables for which data were sought and any assumptions and simplifications made. | 5 |
| Critical appraisal of individual sources of evidence§ | 12 | If done, provide a rationale for conducting a critical appraisal of included sources of evidence; describe the methods used and how this information was used in any data synthesis (if appropriate). | 4 and appendix 4 |
| Synthesis of results | 13 | Describe the methods of handling and summarizing the data that were charted. | 5 |
| **RESULTS** | | | |
| Selection of sources of evidence† | 14 | Give number of sources of evidence screened, assessed for eligibility, and included in the review, with reasons for exclusions at each stage, ideally using a flow diagram. | 7 and figure 1 |
| Characteristics of sources of evidence | 15 | For each source of evidence, present characteristics for which data were charted and provide the citations. | 8-9 |
| Critical appraisal within sources of evidence | 16 | If done, present data on critical appraisal of included sources of evidence (see item 12). | 4 and appendix 4 |
| Results of  individual sources of evidence | 17 | For each included source of evidence, present the relevant data that were charted that relate to the review questions and objectives. | 8-10 |
| Synthesis of results | 18 | Summarize and/or present the charting results as they relate to the review questions and objectives. | 8-12 |
| **DISCUSSION** | | | |
| Summary of evidence | 19 | Summarize the main results (including an overview of concepts, themes, and types of evidence available), link to the review questions and objectives, and consider the relevance to key groups. | 15 |
| Limitations | 20 | Discuss the limitations of the scoping review process. | 17 |
| Conclusions | 21 | Provide a general interpretation of the results with respect to the review questions and objectives, as well as potential implications and/or next steps. | 18 |
| **FUNDING** | | | |
| Funding | 22 | Describe sources of funding for the included sources of evidence, as well as sources of funding for the scoping review. Describe the role of the funders of the scoping review. | 19 |

JBI = Joanna Briggs Institute; PRISMA-ScR = Preferred Reporting Items for Systematic reviews and Meta-Analyses extension for Scoping Reviews.

* Where *sources of evidence* (see second footnote) are compiled from, such as bibliographic databases, social media

platforms, and Web sites.

† A more inclusive/heterogeneous term used to account for the different types of evidence or data sources (e.g., quantitative and/or qualitative research, expert opinion, and policy documents) that may be eligible in a scoping review as opposed to only studies. This is not confused with *information sources* (see first footnote).

‡ The frameworks by Arksey and O’Malley (6) and Levac and colleagues (7) and the JBI guidance (4, 5) refer to the process of data extraction in a scoping review as data charting*.*

§ The process of systematically examining research evidence to assess its validity, results, and relevance before using it to inform a decision. This term is used for items 12 and 19 instead of "risk of bias" (which is more applicable to systematic reviews of interventions) to include and acknowledge the various sources of evidence that may be used in a scoping review (e.g., quantitative and/or qualitative research, expert opinion, and policy document).

*From:* Tricco AC, Lillie E, Zarin W, O'Brien KK, Colquhoun H, Levac D, et al. PRISMA Extension for Scoping Reviews (PRISMAScR): Checklist and Explanation. Ann Intern Med.

# Appendix 3

# Search Strategy and Results

**1.Web of Science (searched March 19, 2023)**

| # | Searches | Results |
| --- | --- | --- |
| Related to vaccination/immunization coverage and challenge | | |
| 1 | (((((((TS=(vaccin*)) OR TS=(immuni*)) OR TS=(vaccin* coverage)) OR TS=(vaccin* uptake)) OR TS=(vaccin* rate)) OR TS =(immuni* coverage)) OR TS=(immuni* uptake)) OR TS=(immuni* rate) | 1,960,989 |
| 2 | (((((((((TS=(challenge*)) OR TS=(barrier*)) OR TS=(problem*)) OR TS=(issue*)) OR TS=(reason*)) OR TS=(root*)) OR TS=(determinant*)) OR TS=(hinder*)) OR TS=(constraint*)) OR TS=(factor*) | 29,423,785 |
| 3 | TI=(China) | 579,588 |
| 4 | #1 AND #2 AND #3 | 2,281 |
| 5 | 2013-2023 (Publication Years) and English or Chinese (Languages) | 2,272 |
| Related to non-NIP vaccine immunization coverage and challenge | | |
| 1 | (((((((((((TS=(non-NIP vaccin*)) OR TS=(non immuni* program* vaccin*)) OR TS=(out-of-pocket vaccin*)) OR TS=(class 2 vaccin*)) OR TS=(category 2 vaccin*)) OR TS=(optional vaccin*)) OR TS=(non-EPI vaccin*)) OR TS=(non expanded program* immun* vaccin*)) OR TS=(non immuni* program* vaccin* coverage)) OR TS=(non immuni* program* vaccin* uptake)) OR TS=(non immuni* program* vaccin* rate)） | 20,759 |
| 2 | (((((((((TS=(challenge*)) OR TS=(barrier*)) OR TS=(problem*)) OR TS=(issue*)) OR TS=(reason*)) OR TS=(root*)) OR TS=(determinant*)) OR TS=(hinder*)) OR TS=(constraint*)) OR TS=(factor*) | 29,423,785 |
| 3 | TS=(China) | 1,631,953 |
| 4 | #1 AND #2 AND #3 | 148 |
| 5 | 2013 -2012 (Publication Years) and English or Chinese (Languages) | 131 |
| Related to HPV vaccination coverage and challenge | | |
| 1 | (((((((((TS=(papillomavirus vaccin*)) OR TS=(HPV vaccni*)) OR TS=(HPV immuni*)) OR TS=(human papillomavirus vaccin*)) OR TS=(Cecolin)) OR TS=(Cervarix)) OR TS=(Gardasil)) OR TS=(HPV vaccni* coverage)) OR TS=(HPV vaccni* rate)) OR TS=(HPV vaccni* uptake) | [25,89](https://www.webofscience.com/wos/alldb/summary/a05faf4b-5510-4511-bb57-6e91372b142a-7a0a48f5/relevance/1)0 |
| 2 | (((((((((TS=(challenge*)) OR TS=(barrier*)) OR TS=(problem*)) OR TS=(issue*)) OR TS=(reason*)) OR TS=(root*)) OR TS=(determinant*)) OR TS=(hinder*)) OR TS=(constraint*)) OR TS=(factor*) | [29,423,785](https://www.webofscience.com/wos/alldb/summary/5d00d04d-4e15-4023-9509-158fd1e4272c-7a219dd8/relevance/1) |
| 3 | TS=(China) | 1,631,953 |
| 4 | #1 AND #2 AND #3 | 218 |
| 5 | 2013 -2012 (Publication Years) and English or Chinese (Languages) | 193 |
| Related to Rotavirus vaccination coverage and challenge | | |
| 1 | ((((((((TS=(Rotavirus Vaccin*)) OR TS=(RotaTeq)) OR TS=(LLR vaccin*)) OR TS=(Pentavalent rotavirus vaccin*)) OR TS=(Lanzhou lamb rotavirus vaccin*)) OR TS=(Rotavirus immun*)) OR TS=(Rotavirus vaccin* coverage)) OR TS=(Rotavirus vaccin* rate)) OR TS=(Rotavirus vaccin* uptake) | 25,890 |
| 2 | (((((((((TS=(challenge*)) OR TS=(barrier*)) OR TS=(problem*)) OR TS=(issue*)) OR TS=(reason*)) OR TS=(root*)) OR TS=(determinant*)) OR TS=(hinder*)) OR TS=(constraint*)) OR TS=(factor*) | 29,423,785 |
| 3 | TS=(China) | 1,631,953 |
| 4 | #1 AND #2 AND #3 | 21 |
| 5 | 2013 -2012 (Publication Years) and English or Chinese (Languages) | 19 |
| Related to Hib vaccination coverage and challenge | | |
| 1 | ((((((TS=(Haemophilus influenzae type b vaccin*)) OR TS=(Hib vaccin*)) OR TS=(Haemophilus Type b Conjugate Vaccin*)) OR TS=(Hib immuni*)) OR TS=(Hib vaccin* coverage)) OR TS=(Hib vaccin* uptake)) OR TS=(Hib vaccin* rate) | 7,122 |
| 2 | (((((((((TS=(challenge*)) OR TS=(barrier*)) OR TS=(problem*)) OR TS=(issue*)) OR TS=(reason*)) OR TS=(root*)) OR TS=(determinant*)) OR TS=(hinder*)) OR TS=(constraint*)) OR TS=(factor*) | 29,423,785 |
| 3 | TS=(China) | 1,631,953 |
| 4 | #1 AND #2 AND #3 | 20 |
| 5 | 2013 -2012 (Publication Years) and English or Chinese (Languages) | 13 |
| Related to PCV vaccination coverage and challenge | | |
| 1 | ((((((TS=(Pneumococcal vaccin*)) OR TS=(PCV vaccin*)) OR TS=(23-valent Pneumococcal polysaccharide vaccin*)) OR TS=(13-Valent Pneumococcal Polysaccharide Conjugate Vaccin*)) OR TS=(PCV vaccin* coverage)) OR TS=(PCV vaccin* uptake)) OR TS=(PCV vaccin* rate) | 24,509 |
| 2 | (((((((((TS=(challenge*)) OR TS=(barrier*)) OR TS=(problem*)) OR TS=(issue*)) OR TS=(reason*)) OR TS=(root*)) OR TS=(determinant*)) OR TS=(hinder*)) OR TS=(constraint*)) OR TS=(factor*) | 29,423,785 |
| 3 | TS=(China) | 1,631,953 |
| 4 | #1 AND #2 AND #3 | 40 |
| 5 | 2013 -2012 (Publication Years) and English or Chinese (Languages) | 36 |

**2.PubMed (searched March 19, 2023)**

| Related to vaccination/immunization coverage and challenge | | |
| --- | --- | --- |
| 1 | (((((((vaccin*[MeSH Terms]) OR (immuni*[Title/Abstract])) OR (vaccin* coverage[Title/Abstract])) OR (vaccin* uptake[Title/Abstract])) OR (vaccin* rate[Title/Abstract])) OR (immuni* coverage[Title/Abstract])) OR (immuni* uptake[Title/Abstract])) OR (immuni* rate[Title/Abstract]) | 632,294 |
| 2 | (("challenge*"[Title/Abstract] OR "barrier*"[Title/Abstract] OR "problem*"[Title/Abstract] OR "issue*"[Title/Abstract] OR "reason*"[Title/Abstract] OR "root*"[Title/Abstract] OR "determinant*"[Title/Abstract] OR "hinder*"[Title/Abstract]) OR (factor*[Title/Abstract])) OR (constraint*[Title/Abstract]) | 7,403,181 |
| 3 | China[Title/Abstract] | 282,903 |
| 4 | #1 AND #2 AND #3 | 2,972 |
| 5 | Filters: English, from 2013 - 2023 | 2,329 |
| Related to non-NIP vaccine immunization coverage and challenge | | |
| 1 | (((((((non-NIP vaccine*) OR (non immuni* program* vaccine*)OR (self-paid vaccine*)OR (out-of-pocket vaccine*)OR (category 2 vaccine*) OR (class 2 vaccine*) OR (optional vaccine*)) OR (non-EPI vaccin*[Title/Abstract])) ) OR (non expanded program* immun* vaccin*[Title/Abstract])) OR (non immuni* program* vaccin* coverage[Title/Abstract])) OR (non immuni* program* vaccin* uptake[Title/Abstract])) OR (non immuni* program* vaccin* rate[Title/Abstract]) | [20,35](https://pubmed.ncbi.nlm.nih.gov/?term=%28non-NIP+vaccine%2A%29+OR+%28non+immunization+programme+vaccine%2A%29OR+%28self-paid+vaccine%2A%29OR+%28out-of-pocket+vaccine%2A%29OR+%28category+2+vaccine%2A%29+OR+%28class+2+vaccine%2A%29+OR+%28optional+vaccine%2A%29&sort=)2 |
| 2 | (("challenge*"[Title/Abstract] OR "barrier*"[Title/Abstract] OR "problem*"[Title/Abstract] OR "issue*"[Title/Abstract] OR "reason*"[Title/Abstract] OR "root*"[Title/Abstract] OR "determinant*"[Title/Abstract] OR "hinder*"[Title/Abstract]) OR (factor*[Title/Abstract])) OR (constraint*[Title/Abstract]) | 7,403,181 |
| 3 | China[Title/Abstract] | 282,903 |
| 4 | #1 AND #2 AND #3 | 202 |
| 5 | Filters: English, from 2013 - 2023 | 187 |
| Related to HPV vaccination coverage and challenge | | |
| 1 | (((((((((Papillomavirus Vaccin*[MeSH Terms]) OR (HPV vaccin*[Title/Abstract])) OR (human papillomavirus vaccin*[Title/Abstract])) OR (Cecolin[Title/Abstract])) OR (Cervarix[Title/Abstract])) OR (Gardasil[Title/Abstract])) OR (HPV immuni*) ) OR (HPV vaccni* coverage[Title/Abstract])) OR (HPV vaccni* rate[Title/Abstract])) OR (HPV vaccni* uptake[Title/Abstract]) | 15,474 |
| 2 | (("challenge*"[Title/Abstract] OR "barrier*"[Title/Abstract] OR "problem*"[Title/Abstract] OR "issue*"[Title/Abstract] OR "reason*"[Title/Abstract] OR "root*"[Title/Abstract] OR "determinant*"[Title/Abstract] OR "hinder*"[Title/Abstract]) OR (factor*[Title/Abstract])) OR (constraint*[Title/Abstract]) | 7,403,181 |
| 3 | China[Title/Abstract] | 282,903 |
| 4 | #1 AND #2 AND #3 | 143 |
| 5 | Filters: English, from 2013 - 2023 | 127 |
| Related to PCV vaccination coverage and challenge | | |
| 1 | (((((((Pneumococcal Vaccin*[MeSH Terms]) OR (PCV vaccin*[Title/Abstract])) OR (23-valent Pneumococcal polysaccharide vaccin*[Title/Abstract])) OR (13-Valent Pneumococcal Polysaccharide conjugate vaccin*[Title/Abstract])) OR (PCV vaccin* coverage)) OR (PCV vaccin* uptake)) OR (PCV vaccin* rate)) OR (PCV immuni*[Title/Abstract]) | 9199 |
| 2 | (("challenge*"[Title/Abstract] OR "barrier*"[Title/Abstract] OR "problem*"[Title/Abstract] OR "issue*"[Title/Abstract] OR "reason*"[Title/Abstract] OR "root*"[Title/Abstract] OR "determinant*"[Title/Abstract] OR "hinder*"[Title/Abstract]) OR (factor*[Title/Abstract])) OR (constraint*[Title/Abstract]) | 7,403,181 |
| 3 | China[Title/Abstract] | 282,903 |
| 4 | #1 AND #2 AND #3 | 38 |
| 5 | Filters: English, from 2013 - 2023 | 32 |
| Related to Hib vaccination coverage and challenge | | |
| 1 | ((((((Haemophilus influenzae type b*[MeSH Terms]) OR (Haemophilus Type b Conjugate Vaccin*[Title/Abstract])) OR (Hib vaccin*[Title/Abstract])) OR (Hib immuni*[Title/Abstract])) OR (Hib vaccin* coverage[Title/Abstract])) OR (Hib vaccin* uptake[Title/Abstract])) OR (Hib vaccin* rate[Title/Abstract]) | 2,374 |
| 2 | (("challenge*"[Title/Abstract] OR "barrier*"[Title/Abstract] OR "problem*"[Title/Abstract] OR "issue*"[Title/Abstract] OR "reason*"[Title/Abstract] OR "root*"[Title/Abstract] OR "determinant*"[Title/Abstract] OR "hinder*"[Title/Abstract]) OR (factor*[Title/Abstract])) OR (constraint*[Title/Abstract]) | 7,403,181 |
| 3 | China[Title/Abstract] | 282,903 |
| 4 | #1 AND #2 AND #3 | 10 |
| 5 | Filters: English, from 2013 - 2023 | 5 |
| Related to Rotavirus vaccination coverage and challenge | | |
| 1 | (((((((Rotavirus Vaccin*[MeSH Terms]) OR (RotaTeq[Title/Abstract])) OR (Pentavalent rotavirus vaccin*[Title/Abstract])) OR (Lanzhou lamb rotavirus vaccin*[Title/Abstract])) OR (LLR vaccin*[Title/Abstract])) OR (Rotavirus vaccin* coverage)) OR (Rotavirus vaccin* rate)) OR (Rotavirus vaccin* uptake) | 3,482 |
| 2 | (("challenge*"[Title/Abstract] OR "barrier*"[Title/Abstract] OR "problem*"[Title/Abstract] OR "issue*"[Title/Abstract] OR "reason*"[Title/Abstract] OR "root*"[Title/Abstract] OR "determinant*"[Title/Abstract] OR "hinder*"[Title/Abstract]) OR (factor*[Title/Abstract])) OR (constraint*[Title/Abstract]) | 7,403,181 |
| 3 | China[Title/Abstract] | 282,903 |
| 4 | #1 AND #2 AND #3 | 17 |
| 5 | Filters: English, from 2013 - 2023 | 12 |

**3.Scopus (searched March 19, 2023)**

| Related to vaccination/immunization coverage and challenge | | |
| --- | --- | --- |
| 1 | TITLE-ABS-KEY (immuni* OR vaccin* OR "vaccin* coverage" OR "vaccin* uptake" OR "vaccin* rate" OR "immuni* coverage" OR "immuni* uptake" OR "immuni* rate") | 1,185,949 |
| 2 | TITLE-ABS-KEY (challenge* OR barrier* OR problem* OR issue* OR reason* OR root* OR determinant* OR hinder* OR factor* OR constraint* ) | 23,860,988 |
| 3 | TITLE (China ) | 465,376 |
| 4 | #1 AND #2 AND #3 | 2,098 |
| 5 | PUBYEAR > 2012 AND PUBYEAR < 2024 AND ( LIMIT-TO ( LANGUAGE,"English" ) OR LIMIT-TO ( LANGUAGE,"Chinese" ) ) | 1,715 |
| Related to non-NIP vaccine immunization coverage and challenge | | |
| 1 | ALL ( "non-NIP vaccin*" OR "non immunization program vaccine*" OR "self-paid vaccine*" OR "out-of-pocket vaccine*" OR "category 2 vaccine*" OR "class 2 vaccin*" OR "optional vaccin*" OR "non-EPI vaccin*" OR "non expanded program* immun* vaccin*" OR "non immuni* program* vaccin* coverage" OR "non immuni* program* vaccin* uptake" OR "non immuni* program* vaccin* rate" ) | 288 |
| 2 | TITLE-ABS-KEY ( challenge* OR barrier* OR problem* OR issue* OR reason* OR root* OR determinant* OR hinder* OR factor* OR constraint* ) | 23,860,988 |
| 3 | TITLE (China ) | 465,376 |
| 4 | #1 AND #2 AND #3 | 85 |
| 5 | LIMIT-TO (PUBYEAR,2013-2023) AND (LIMIT-TO (LANGUAGE,"English") OR LIMIT-TO ( LANGUAGE,"Chinese" ) | 74 |
| Related to HPV vaccination coverage and challenge | | |
| 1 | TITLE-ABS ("Papillomavirus Vaccin*" OR "HPV vaccin*" OR "human papillomavirus vaccin*" OR "HPV immuni*" OR "Cecolin" OR "Cervarix" OR "Gardasil" OR "HPV vaccni* coverage" OR "HPV vaccni* rate" OR "HPV vaccni* uptake" ) | 12,802 |
| 2 | TITLE-ABS-KEY ( challenge* OR barrier* OR problem* OR issue* OR reason* OR root* OR determinant* OR hinder* OR factor* OR constraint* ) | 23,860,988 |
| 3 | TITLE (China ) | 465,376 |
| 4 | #1 AND #2 AND #3 | 119 |
| 5 | LIMIT-TO (PUBYEAR,2013-2023) AND (LIMIT-TO (LANGUAGE,"English") OR LIMIT-TO ( LANGUAGE,"Chinese" ) | 112 |
| Related to PCV vaccination coverage and challenge | | |
| 1 | TITLE-ABS-KEY("Pneumococcal vaccin*" OR "PCV vaccin*" OR "23-valent Pneumococcal polysaccharide vaccin*" OR "13-Valent Pneumococcal Polysaccharide Conjugate Vaccin*" OR "PCV vaccin* coverage" OR "PCV vaccin* uptake" OR "PCV vaccin* rate") | 11,958 |
| 2 | TITLE-ABS-KEY ( challenge* OR barrier* OR problem* OR issue* OR reason* OR root* OR determinant* OR hinder* OR factor* OR constraint* ) | 23,860,988 |
| 3 | TITLE (China ) | 465,376 |
| 4 | #1 AND #2 AND #3 | 26 |
| 5 | LIMIT-TO (PUBYEAR,2013-2023) AND (LIMIT-TO (LANGUAGE,"English") OR LIMIT-TO ( LANGUAGE,"Chinese" ) | 24 |
| Related to Hib vaccination coverage and challenge | | |
| 1 | TITLE-ABS ( "Haemophilus influenzae type b*" OR "Haemophilus Type b Conjugate Vaccin*" OR "Hib vaccin*" OR "Hib immuni*" OR "Hib vaccin* coverage" OR "Hib vaccin* uptake" OR "Hib vaccin* rate" ) | 5,726 |
| 2 | TITLE-ABS-KEY ( challenge* OR barrier* OR problem* OR issue* OR reason* OR root* OR determinant* OR hinder* OR factor* OR constraint* ) | 23,860,988 |
| 3 | TITLE (China ) | 465,376 |
| 4 | #1 AND #2 AND #3 | 12 |
| 5 | LIMIT-TO (PUBYEAR,2013-2023) AND (LIMIT-TO (LANGUAGE,"English") OR LIMIT-TO ( LANGUAGE,"Chinese" ) | 7 |
| Related to Rotavirus vaccination coverage and challenge | | |
| 1 | TITLE-ABS ( "Rotavirus Vaccin*"  OR  "RotaTeq"  OR  "Pentavalent rotavirus vaccin*"  OR  "Lanzhou lamb rotavirus vaccin*"  OR  "LLR vaccin*"  OR  "Rotavirus immun*"  OR  "Rotavirus vaccin* coverage"  OR  "Rotavirus vaccin* rate"  OR  "Rotavirus vaccin* uptake | 4,208 |
| 2 | TITLE-ABS-KEY ( challenge* OR barrier* OR problem* OR issue* OR reason* OR root* OR determinant* OR hinder* OR factor* OR constraint* ) | 23,860,988 |
| 3 | TITLE (China ) | 465,376 |
| 4 | #1 AND #2 AND #3 | 12 |
| 5 | LIMIT-TO (PUBYEAR,2013-2023) AND (LIMIT-TO (LANGUAGE,"English") OR LIMIT-TO ( LANGUAGE,"Chinese" ) | 9 |

**4.Embase (searched March 19, 2023)**

| # | Searches | Results |
| --- | --- | --- |
| Related to vaccination/immunization coverage and challenge | | |
| 1 | 'vaccine'/exp OR 'immunization'/exp OR 'vaccin* coverage':ti,ab,kw OR 'vaccin* uptake':ti,ab,kw OR 'vaccin* rate':ti,ab,kw OR 'immuni* coverage':ti,ab,kw OR 'immuni* uptake':ti,ab,kw OR 'immuni* rate':ti,ab,kw | 589,763 |
| 2 | challenge* OR barrier*:ti,ab,kw OR problem*:ti,ab,kw OR issue*:ti,ab,kw OR reason*:ti,ab,kw OR root*:ti,ab,kw OR determinant*:ti,ab,kw OR hinder*:ti,ab,kw OR factor*:ti,ab,kw OR constraint*:ti,ab,kw | 5,448,485 |
| 3 | china:ti | 135,130 |
| 4 | #1 AND #2 AND #3 | 697 |
| 5 | (2013:py OR 2014:py OR 2015:py OR 2016:py OR 2017:py OR 2018:py OR 2019:py OR 2020:py OR 2021:py OR 2022:py OR 2023:py) | 615 |
| Related to non-NIP vaccine immunization coverage and challenge | | |
| 1 | 'non-nip vaccin*' OR 'non immuni* program* vaccin*' OR 'non immunization program vaccin*' OR 'out-of-pocket vaccin*' OR 'class 2 vaccin*' OR 'category 2 vaccin*' OR 'optional vaccin*' OR 'non-epi vaccin*' OR 'non expanded program* immun* vaccin*' OR 'non immuni* program* vaccin* coverage' OR 'non immuni* program* vaccin* uptake' OR 'non immuni* program* vaccin* rate' | 93 |
| 2 | china:ti | 135,130 |
| 3 | #1 AND #2 AND #3 | 25 |
| Related to HPV vaccination coverage and challenge | | |
| 1 | 'human papilloma virus vaccine'/exp OR 'hpv vaccni':ti,ab,kw OR 'hpv immuni*':ti,ab,kw OR 'human papillomavirus vaccin*':ti,ab,kw OR 'hpv vaccni* uptake':ti,ab,kw OR cecolin:ti,ab,kw OR cervarix:ti,ab,kw OR gardasil:ti,ab,kw OR 'hpv vaccni* coverage':ti,ab,kw OR 'hpv vaccni* rate':ti,ab,kw | 18,083 |
| 2 | challenge* OR barrier*:ti,ab,kw OR problem*:ti,ab,kw OR issue*:ti,ab,kw OR reason*:ti,ab,kw OR root*:ti,ab,kw OR determinant*:ti,ab,kw OR hinder*:ti,ab,kw OR factor*:ti,ab,kw OR constraint*:ti,ab,kw | 5,448,485 |
| 3 | china:ti | 135,130 |
| 4 | #1 AND #2 AND #3 | 66 |
| 5 | (2013:py OR 2014:py OR 2015:py OR 2016:py OR 2017:py OR 2018:py OR 2020:py OR 2021:py OR 2022:py OR 2023:py) | 54 |
| Related to Rotavirus vaccination coverage and challenge | | |
| 1 | 'rotavirus vaccine'/exp OR 'pentavalent rotavirus vaccin*':ti,ab,kw OR 'lanzhou lamb rotavirus vaccin*':ti,ab,kw OR 'rotavirus immun*':ti,ab,kw OR 'rotavirus vaccin* coverage':ti,ab,kw OR rotateq:ti,ab,kw OR 'llr vaacin*':ti,ab,kw OR 'rotavirus vaccin* rate':ti,ab,kw OR 'rotavirus vaccin* uptake':ti,ab,kw | 6,710 |
| 2 | challenge* OR barrier*:ti,ab,kw OR problem*:ti,ab,kw OR issue*:ti,ab,kw OR reason*:ti,ab,kw OR root*:ti,ab,kw OR determinant*:ti,ab,kw OR hinder*:ti,ab,kw OR factor*:ti,ab,kw OR constraint*:ti,ab,kw | 5,448,485 |
| 3 | china:ti | 135,130 |
| 4 | #1 AND #2 AND #3 | 12 |
| 5 | (2015:py OR 2018:py OR 2019:py OR 2021:py OR 2022:py) | 7 |
| Related to Hib vaccination coverage and challenge | | |
| 1 | 'haemophilus influenzae type b vaccine'/exp OR 'hib vaccin*':ti,ab,kw OR 'haemophilus type b conjugate vaccin*':ti,ab,kw OR 'hib immuni*':ti,ab,kw OR 'hib vaccin* uptake':ti,ab,kw OR 'pcv vaccin* uptake':ti,ab,kw OR 'hib vaccin* rate':ti,ab,kw | 6,022 |
| 2 | challenge* OR barrier*:ti,ab,kw OR problem*:ti,ab,kw OR issue*:ti,ab,kw OR reason*:ti,ab,kw OR root*:ti,ab,kw OR determinant*:ti,ab,kw OR hinder*:ti,ab,kw OR factor*:ti,ab,kw OR constraint*:ti,ab,kw | 5,448,485 |
| 3 | china:ti | 135,130 |
| 4 | #1 AND #2 AND #3 | 11 |
| 5 | (2015:py OR 2018:py OR 2019:py OR 2021:py OR 2022:py) | 6 |
| Related to PCV vaccination coverage and challenge | | |
| 1 | 'pneumococcus vaccine'/exp OR 'pcv vaccin*':ti,ab,kw OR '23-valent pneumococcal polysaccharide vaccin*':ti,ab,kw OR '13-valent pneumococcal polysaccharide conjugate vaccin*':ti,ab,kw OR 'pcv vaccin* coverage':ti,ab,kw OR 'pcv vaccin* uptake':ti,ab,kw OR 'pcv vaccin* rate':ti,ab,kw OR 'pcv immuni*':ti,ab,kw | 23,168 |
| 2 | challenge* OR barrier*:ti,ab,kw OR problem*:ti,ab,kw OR issue*:ti,ab,kw OR reason*:ti,ab,kw OR root*:ti,ab,kw OR determinant*:ti,ab,kw OR hinder*:ti,ab,kw OR factor*:ti,ab,kw OR constraint*:ti,ab,kw | 5,448,485 |
| 3 | china:ti | 135,130 |
| 4 | #1 AND #2 AND #3 | 31 |
| 5 | (2014:py OR 2015:py OR 2016:py OR 2018:py OR 2019:py OR 2020:py OR 2021:py OR 2022:py) | 25 |

**5.CNKI (searched March 19, 2023)**

| # | Searches | Results |
| --- | --- | --- |
| Related to non-NIP vaccine immunization coverage and challenge | | |
| 1 | TKA='二类疫苗' + '自费疫苗' + '非免疫规划疫苗'+'非EPI疫苗' AND TKA% '覆盖' +'接种率' + '接种量' + '接种差异' +'接种公平' + '接种不公平' AND FT% '问题'+'不足' + '劣势'+ '阻碍' + '障碍' + '限制' + '制约' + '因素' + '要素' NOT TI='指南'+'共识'+'临床'+'研制'+'抗体'+'新冠' +'新型冠状病毒'+'COVID' Date:2013-2023 | 182 |
| Related to HPV vaccination coverage and challenge | | |
| 1 | TKA='HPV疫苗' + '宫颈癌疫苗' + '人乳头瘤病毒疫苗' AND TKA% '覆盖' +'接种率' + '接种量' + '接种差异' +'接种公平' + '接种不公平' AND FT% '问题'+'不足' + '劣势'+ '阻碍' + '障碍' + '限制' + '制约' + '因素' + '要素' NOT TI='指南'+'共识'+'临床'+'研制'+'抗体'+'新冠' +'新型冠状病毒'+'COVID' Date:2013-2023 | 173 |
| Related to Rotavirus vaccination coverage and challenge | | |
| 1 | TKA='轮状疫苗'+'轮状病毒疫苗' AND TKA% '覆盖' +'接种率' + '接种量' + '接种差异' +'接种公平' + '接种不公平' AND FT% '问题'+'不足' + '劣势'+ '阻碍' + '障碍' + '限制' + '制约' + '因素' + '要素' NOT TI='指南'+'共识'+'临床'+'研制'+'抗体'+'新冠' +'新型冠状病毒'+'COVID' Date:2013-2023 | 69 |
| Related to Hib vaccination coverage and challenge | | |
| 1 | TKA='Hib疫苗' + 'b型流感嗜血杆菌'*'疫苗' AND TKA% '覆盖' +'接种率' + '接种量' + '接种差异' +'接种公平' + '接种不公平' AND FT% '问题'+'不足' + '劣势'+ '阻碍' + '障碍' + '限制' + '制约' + '因素' + '要素' NOT TI='指南'+'共识'+'临床'+'研制'+'抗体'+'新冠' +'新型冠状病毒'+'COVID' Date:2013-2023 | 60 |
| Related to PCV vaccination coverage and challenge | | |
| 1 | TKA='PCV疫苗' + '肺炎疫苗' + '肺炎球菌'+'肺炎链球菌疫苗' AND TKA% '覆盖' +'接种率' + '接种量' + '接种差异' +'接种公平' + '接种不公平' AND FT% '问题'+'不足' + '劣势'+ '阻碍' + '障碍' + '限制' + '制约' + '因素' + '要素' NOT TI='指南'+'共识'+'临床'+'研制'+'抗体'+'新冠' +'新型冠状病毒'+'COVID' Date:2013-2023 | 166 |

**6.Wan Fang Database (searched March 19, 2023)**

| # | Searches | Results |
| --- | --- | --- |
| Related to non-NIP vaccine immunization coverage and challenge | | |
| 1 | (摘要:(二类疫苗 or 自费疫苗 or 非免疫规划疫苗 or 非EPI疫苗) and 摘要:(覆盖 or 接种率 or 接种量 or 接种差异 or 接种公平 or 接种不公平) and 全部:(问题 or 不足 or 劣势 or 阻碍 or 障碍 or 限制 or 制约 or 因素 or 要素) not 题名:("临床 or 指南 or 共识 or 研制 or 抗体 or 新冠 or 新型冠状病毒 or COVID")) and Date:2013-2023 | 217 |
| Related to HPV vaccination coverage and challenge | | |
| 1 | （中英文扩展）： (摘要:(HPV疫苗 + 宫颈癌疫苗 + 人乳头瘤病毒疫苗) and 摘要:(覆盖 or 接种率 or 接种量 or 接种差异 or 接种公平 or 接种不公平) and 全部:(问题 or 不足 or 劣势 or 阻碍 or 障碍 or 限制 or 制约 or 因素 or 要素) not 题名或关键词:("临床 or 指南 or 共识 or 研制 or 抗体 or 新冠 or 新型冠状病毒 or COVID")) and Date:2013-2023 | 286 |
| Related to Rotavirus vaccination coverage and challenge | | |
| 1 | (摘要:(轮状疫苗 or 轮状病毒疫苗) and 摘要:(覆盖 or 接种率 or 接种量 or 接种差异 or 接种公平 or 接种不公平) and 全部:(问题 or 不足 or 劣势 or 阻碍 or 障碍 or 限制 or 制约 or 因素 or 要素) not 题名或关键词:("临床 or 指南 or 共识 or 研制 or 抗体 or 新冠 or 新型冠状病毒 or COVID")) and Date:2013-2023 | 53 |
| Related to Hib vaccination coverage and challenge | | |
| 1 | (摘要:(Hib疫苗 or b型流感嗜血杆菌疫苗) and 摘要:(覆盖 or 接种率 or 接种量 or 接种差异 or 接种公平 or 接种不公平) and 全部:(问题 or 不足 or 劣势 or 阻碍 or 障碍 or 限制 or 制约 or 因素 or 要素) not 题名或关键词:("临床 or 指南 or 共识 or 研制 or 抗体 or 新冠 or 新型冠状病毒 or COVID")) and Date:2013-2023 | 41 |
| Related to PCV vaccination coverage and challenge | | |
| 1 | (摘要:(PCV疫苗 or 肺炎球菌疫苗) and 题名或关键词:(覆盖 or 接种率 or 接种量 or 接种差异 or 接种公平 or 接种不公平) and 全部:(问题 or 不足 or 劣势 or 阻碍 or 障碍 or 限制 or 制约 or 因素 or 要素) not 题名或关键词:("临床 or 指南 or 共识 or 研制 or 抗体 or 新冠 or 新型冠状病毒 or COVID")) and Date:2013-2023 | 84 |

**7.China Science and Technology Journal Database, (searched March 19, 2023)**

| # | Searches | Results |
| --- | --- | --- |
| Related to non-NIP vaccine immunization coverage and challenge | | |
| 1 | ((((((摘要=二类疫苗 OR 摘要=自费疫苗) OR 摘要=非免疫规划疫苗) OR 摘要=非EPI疫苗) AND (((((摘要=覆盖 OR 摘要=接种率) OR 摘要=接种量) OR 摘要=接种差异) OR 摘要=接种公平) OR 摘要=接种不公平)) AND ((((((((任意字段=问题 OR 任意字段=不足) OR 任意字段=劣势) OR 任意字段=阻碍) OR 任意字段=障碍) OR 任意字段=限制) OR 任意字段=制约) OR 任意字段=因素) OR 任意字段=要素)) AND ( NOT (((((((题名或关键词=临床 OR 题名或关键词=指南) OR 题名或关键词=共识) OR 题名或关键词=研制) OR 题名或关键词=抗体) OR 题名或关键词=新冠) OR 题名或关键词=新型冠状病毒) OR 题名或关键词=COVID))) AND (years:[2013 TO 2023]) | 86 |
| Related to HPV vaccination coverage and challenge | | |
| 1 | (((((摘要=HPV疫苗 OR 摘要=宫颈癌疫苗) OR 摘要=人乳头瘤病毒疫苗) AND (((((摘要=覆盖 OR 摘要=接种率) OR 摘要=接种量) OR 摘要=接种差异) OR 摘要=接种公平) OR 摘要=接种不公平)) AND ((((((((任意字段=问题 OR 任意字段=不足) OR 任意字段=劣势) OR 任意字段=阻碍) OR 任意字段=障碍) OR 任意字段=限制) OR 任意字段=制约) OR 任意字段=因素) OR 任意字段=要素)) AND ( NOT (((((((题名或关键词=临床 OR 题名或关键词=指南) OR 题名或关键词=共识) OR 题名或关键词=研制) OR 题名或关键词=抗体) OR 题名或关键词=新冠) OR 题名或关键词=新型冠状病毒) OR 题名或关键词=COVID))) AND (years:[2013 TO 2023]) | 143 |
| Related to Rotavirus vaccination coverage and challenge | | |
| 1 | ((((摘要=轮状疫苗 OR摘要=轮状病毒疫苗) AND (((((摘要=覆盖 OR 摘要=接种率) OR 摘要=接种量) OR 摘要=接种差异) OR 摘要=接种公平) OR 摘要=接种不公平)) AND ((((((((任意字段=问题 OR 任意字段=不足) OR 任意字段=劣势) OR 任意字段=阻碍) OR 任意字段=障碍) OR 任意字段=限制) OR 任意字段=制约) OR 任意字段=因素) OR 任意字段=要素)) AND ( NOT (((((((题名或关键词=临床 OR 题名或关键词=指南) OR 题名或关键词=共识) OR 题名或关键词=研制) OR 题名或关键词=抗体) OR 题名或关键词=新冠) OR 题名或关键词=新型冠状病毒) OR 题名或关键词=COVID))) AND (years:[2013 TO 2023]) | 11 |
| Related to Hib vaccination coverage and challenge | | |
| 1 | ((((摘要=Hib疫苗 OR 摘要=b型流感嗜血杆菌疫苗) AND (((((摘要=覆盖 OR 摘要=接种率) OR 摘要=接种量) OR 摘要=接种差异) OR 摘要=接种公平) OR 摘要=接种不公平)) AND ((((((((任意字段=问题 OR 任意字段=不足) OR 任意字段=劣势) OR 任意字段=阻碍) OR 任意字段=障碍) OR 任意字段=限制) OR 任意字段=制约) OR 任意字段=因素) OR 任意字段=要素)) AND ( NOT (((((((题名或关键词=临床 OR 题名或关键词=指南) OR 题名或关键词=共识) OR 题名或关键词=研制) OR 题名或关键词=抗体) OR 题名或关键词=新冠) OR 题名或关键词=新型冠状病毒) OR 题名或关键词=COVID))) AND (years:[2013 TO 2023]) | 25 |
| Related to PCV vaccination coverage and challenge | | |
| 1 | ((((摘要=PCV疫苗 OR 摘要=肺炎球菌疫苗) AND (((((摘要=覆盖 OR 摘要=接种率) OR 摘要=接种量) OR 摘要=接种差异) OR 摘要=接种公平) OR 摘要=接种不公平)) AND ((((((((任意字段=问题 OR 任意字段=不足) OR 任意字段=劣势) OR 任意字段=阻碍) OR 任意字段=障碍) OR 任意字段=限制) OR 任意字段=制约) OR 任意字段=因素) OR 任意字段=要素)) AND ( NOT (((((((题名或关键词=临床 OR 题名或关键词=指南) OR 题名或关键词=共识) OR 题名或关键词=研制) OR 题名或关键词=抗体) OR 题名或关键词=新冠) OR 题名或关键词=新型冠状病毒) OR 题名或关键词=COVID))) AND (years:[2013 TO 2023]) | 38 |

**8.China Biology Medicine (searched March 19, 2023)**

| # | Searches | Results |
| --- | --- | --- |
| Related to non-NIP vaccine immunization coverage and challenge | | |
| 1 | (( "二类疫苗"[摘要:智能] OR "自费疫苗"[摘要:智能] OR "非免疫规划疫苗"[摘要:智能] OR "非EPI疫苗"[摘要:智能]) AND( "覆盖"[摘要:智能] OR "接种率"[摘要:智能] OR "接种量"[摘要:智能] OR "接种差异"[摘要:智能] OR "接种公平"[摘要:智能] OR "接种不公平"[摘要:智能]) AND( "问题"[全部字段:智能] OR "不足"[全部字段:智能] OR "劣势"[全部字段:智能] OR "阻碍"[全部字段:智能] OR "障碍"[全部字段:智能] OR "限制"[全部字段:智能] OR "制约"[全部字段:智能] OR "因素"[全部字段:智能] OR "要素"[全部字段:智能])) NOT( "临床"[标题] OR "指南"[标题] OR "共识"[标题] OR "研制"[标题] OR "抗体"[标题] OR "新冠"[标题] OR "新型冠状病毒"[标题] OR "COVID"[标题]) AND 2013-2023[日期]) | 58 |
| Related to HPV vaccination coverage and challenge | | |
| 1 | (( "HPV疫苗"[摘要:智能] OR "宫颈癌疫苗"[摘要:智能] OR "人乳头瘤病毒疫苗"[摘要:智能])) AND (( "覆盖"[摘要:智能] OR "接种率"[摘要:智能] OR "接种量"[摘要:智能] OR "接种差异"[摘要:智能] OR "接种公平"[摘要:智能] OR "接种不公平"[摘要:智能])) AND (((( "问题"[全部字段:智能] OR "不足"[全部字段:智能] OR "劣势"[全部字段:智能] OR "阻碍"[全部字段:智能] OR "障碍"[全部字段:智能] OR "限制"[全部字段:智能] OR "制约"[全部字段:智能] OR "因素"[全部字段:智能] OR "要素"[全部字段:智能])) NOT( "临床"[标题] OR "指南"[标题] OR "共识"[标题] OR "研制"[标题] OR "抗体"[标题] OR "新冠"[标题] OR "新型冠状病毒"[标题] OR "COVID"[标题])) AND 2013-2023[日期]) | 76 |
| Related to Rotavirus vaccination coverage and challenge | | |
| 1 | (( "轮状疫苗"[摘要:智能] OR "轮状病毒疫苗"[摘要:智能])) AND (( "覆盖"[摘要:智能] OR "接种率"[摘要:智能] OR "接种量"[摘要:智能] OR "接种差异"[摘要:智能] OR "接种公平"[摘要:智能] OR "接种不公平"[摘要:智能])) AND (((( "问题"[全部字段:智能] OR "不足"[全部字段:智能] OR "劣势"[全部字段:智能] OR "阻碍"[全部字段:智能] OR "障碍"[全部字段:智能] OR "限制"[全部字段:智能] OR "制约"[全部字段:智能] OR "因素"[全部字段:智能] OR "要素"[全部字段:智能])) NOT( "临床"[标题] OR "指南"[标题] OR "共识"[标题] OR "研制"[标题] OR "抗体"[标题] OR "新冠"[标题] OR "新型冠状病毒"[标题] OR "COVID"[标题])) AND 2013-2023[日期]) | 24 |
| Related to Hib vaccination coverage and challenge | | |
| 1 | (( "Hib疫苗"[摘要:智能] OR "b型流感嗜血杆菌疫苗"[摘要:智能])) AND (( "覆盖"[标题:智能] OR "接种率"[标题:智能] OR "接种量"[标题:智能] OR "接种差异"[标题:智能] OR "接种公平"[标题:智能] OR "接种不公平"[标题:智能])) AND (((( "问题"[全部字段:智能] OR "不足"[全部字段:智能] OR "劣势"[全部字段:智能] OR "阻碍"[全部字段:智能] OR "障碍"[全部字段:智能] OR "限制"[全部字段:智能] OR "制约"[全部字段:智能] OR "因素"[全部字段:智能] OR "要素"[全部字段:智能])) NOT( "临床"[标题] OR "指南"[标题] OR "共识"[标题] OR "研制"[标题] OR "抗体"[标题] OR "新冠"[标题] OR "新型冠状病毒"[标题] OR "COVID"[标题])) AND 2013-2023[日期]) | 13 |
| Related to PCV vaccination coverage and challenge | | |
| 1 | (( "PCV疫苗"[摘要:智能] OR "肺炎球菌疫苗"[摘要:智能])) AND (( "覆盖"[摘要:智能] OR "接种率"[摘要:智能] OR "接种量"[摘要:智能] OR "接种差异"[摘要:智能] OR "接种公平"[摘要:智能] OR "接种不公平"[摘要:智能])) AND (((( "问题"[全部字段:智能] OR "不足"[全部字段:智能] OR "劣势"[全部字段:智能] OR "阻碍"[全部字段:智能] OR "障碍"[全部字段:智能] OR "限制"[全部字段:智能] OR "制约"[全部字段:智能] OR "因素"[全部字段:智能] OR "要素"[全部字段:智能])) NOT( "临床"[标题] OR "指南"[标题] OR "共识"[标题] OR "研制"[标题] OR "抗体"[标题] OR "新冠"[标题] OR "新型冠状病毒"[标题] OR "COVID"[标题])) AND 2013-2023[日期]) | 7 |

**9.WHO IRIS (Searched March 19, 2023)**

Subjects were limited to “Vaccination coverage” AND languages were limited to “English or Chinese”.44 items were shown and the reviewer will further review the title and abstract.

# Appendix 4

# JBI Critical Appraisal Checklist

Table 1 JBI Critical Appraisal Checklist for analytical cross-sectional studies in 6 articles about vaccine coverage data

| Studies | Were the criteria for inclusion in the sample clearly defined? | Were the study subjects and the setting described in detail? | Was the exposure measured in a valid and reliable way? | Were objective, standard criteria used for measurement of the condition? | Were confounding factors identified? | Were strategies to deal with confounding factors stated? | Were the outcomes measured in a valid and reliable way? | Was appropriate statistical analysis used? |
| --- | --- | --- | --- | --- | --- | --- | --- | --- |
| Yan Li. et al, | Yes | Yes | NA | Yes | Yes | Yes | Yes | Yes |
| Xiaozhen Lai. et al, | Yes | Yes | NA | Yes | Yes | Yes | Yes | Yes |
| Haijun Zhang. et al, | Yes | Yes | NA | Yes | Yes | Yes | Yes | Yes |
| Chenyan Yue. et al, | Yes | Yes | NA | Yes | Yes | Yes | Yes | Yes |
| Liu Yan. et al, | Yes | Yes | NA | Yes | Yes | Yes | Yes | Yes |
| Yifan Song.et al | Yes | Yes | NA | Yes | Yes | Yes | Yes | Yes |

Table 2 JBI Critical Appraisal Checklist for systematic reviews and research syntheses in 3 articles about vaccine coverage data

| Studies | Is the review question clearly and explicitly stated? | Were the inclusion criteria appropriate for the review question? | Was the search strategy appropriate? | Were the sources and resources used to search for studies adequate? | Were the criteria for appraising studies appropriate? | Was critical appraisal conducted by two or more reviewers independently? | Were there methods to minimize errors in data extraction? | Were the methods used to combine studies appropriate? | Was the likelihood of publication bias assessed? | Were recommendations for policy and/or practice supported by the reported data? | Were the specific directives for new research appropriate? |
| --- | --- | --- | --- | --- | --- | --- | --- | --- | --- | --- | --- |
| Wenmin Li. et al, | Yes | Yes | Yes | Yes | Yes | Yes | Yes | Yes | Yes | Yes | No |
| Ya Yang. et al, | Yes | Yes | Yes | Yes | Yes | Yes | Yes | Yes | Yes | Yes | No |
| Weihao Shao. et al, | Yes | Yes | Yes | Yes | Yes | Yes | Yes | Yes | Yes | Yes | Yes |

Table 3 JBI Critical Appraisal Checklist for qualitative research in 19 articles about vaccine coverage barrier

| Studies | Is there congruity between the stated philosophical perspective and the research methodology? | Is there congruity between the research methodology and the research question or objectives? | Is there congruity between the research methodology and the methods used to collect data? | Is there congruity between the research methodology and the representation and analysis of data? | Is there congruity between the research methodology and the interpretation of results? | Are participants, and their voices, adequately represented? | Is the research ethical according to current criteria or, for recent studies, and is there evidence of ethical approval by an appropriate body? | Do the conclusions drawn in the research report flow from the analysis, or interpretation, of the data? |
| --- | --- | --- | --- | --- | --- | --- | --- | --- |
| Zhiyuan Hou. et al, | Yes | Yes | Yes | Yes | Yes | Yes | Yes | Yes |
| Jie Chang. et al, | Yes | Yes | Yes | Yes | Yes | Yes | Yes | Yes |
| Che Deng. et al, | Yes | Yes | Yes | Yes | Yes | Yes | No | Yes |
| Jianli Wang.et al, | Yes | Yes | Yes | Yes | Yes | Yes | Yes | Yes |
| Mingyu Si. et al, | Yes | Yes | Yes | Yes | Yes | Yes | Yes | Yes |
| Xiaozhen Lai. et al, | Yes | Yes | Yes | Yes | Yes | Yes | Yes | Yes |
| Xiangju Yin. et al, | Yes | Yes | Yes | Yes | Yes | Yes | Yes | Yes |
| Jin Duan. et al, | Yes | Yes | Yes | Yes | Yes | Yes | NA | Yes |
| Xuehai Zhang.et al, | Yes | Yes | Yes | Yes | Yes | Yes | NA | Yes |
| Wenchang Wang.et al, | Yes | Yes | Yes | Yes | Yes | Yes | NA | Yes |
| Li Wang.et al, | Yes | Yes | Yes | Yes | Yes | Yes | Yes | Yes |
| Linhong Wang.et al, | Yes | Yes | Yes | Yes | Yes | Yes | Yes | Yes |
| Wen Wang.et al, | Yes | Yes | Yes | Yes | Yes | Yes | NA | Yes |
| Xiaoqian Wang.et al | Yes | Yes | Yes | Yes | Yes | Yes | NA | Yes |
| Bai Yunhua.et al, | Yes | Yes | Yes | Yes | Yes | Yes | NA | Yes |
| Du Wang.et al, | Yes | Yes | Yes | Yes | Yes | Yes | NA | Yes |
| Dan Gong. et al, | Yes | Yes | Yes | Yes | Yes | Yes | Yes | Yes |
| Shiyu Lin. et al, | Yes | Yes | Yes | Yes | Yes | Yes | Yes | Yes |
| Kaiyi Han. et al, | Yes | Yes | Yes | Yes | Yes | Yes | Yes | Yes |
